# Organization of the Auditory Brainstem in a Lizard, *Gekko gecko*. II. Afferent and Efferent Projections of Nuclei of the Lateral Lemniscus and the Torus Semicircularis

**DOI:** 10.1002/cne.70100

**Published:** 2025-10-17

**Authors:** Dawei Han, Yezhong Tang, Jakob Christensen‐Dalsgaard, Wenru Liang, Catherine E. Carr

**Affiliations:** ^1^ Department of Biology University of Maryland College Park Maryland USA; ^2^ Chengdu Institute of Biology Chinese Academy of Sciences Chengdu China; ^3^ Institute of Biology University of Southern Denmark Odense Denmark

**Keywords:** lateral lemniscus, lizard, Neurobiotin (RRID:AB_2336606), Neurolucida (RRID:SCR_001775), superior olive, tonotopy, torus semicircularis, tract tracing

## Abstract

In reptiles, two taxa, lepidosaurs and archosaurs, have ears with thin tympanic membranes that permit sensitive hearing of high‐frequency sounds in air. The organization of their central auditory systems may reflect this increased sensitivity to sound. To understand auditory processing in lepidosaurs further, we used tract tracing techniques to examine the organization and connections of the lemniscal and midbrain auditory nuclei in the Tokay gecko. The nuclei of the lateral lemniscus (LLD) consist of anterior (LLDa), medial (LLDm), and posterior (LLDp) subdivisions and receive ascending projections from the contralateral cochlear nuclei (nucleus angularis and nucleus laminaris) and the ipsilateral dorsal superior olive (SOd). LLD projects to the ventral division of the ipsilateral torus semicircularis (TS). In the Tokay gecko, the central nucleus of the torus (TSC) is large, with two major subdivisions, ventral and dorsal. Caudally, the TSC is fused at the midline, with the dorsal divisions adjoining their contralateral homolog. More rostrally, the TSC bifurcates to form two wings below the tectal ventricles. The ventral division of the TSC receives ascending input from NA/NL, SOd, and the lateral lemniscal complex, while both the dorsal and ventral divisions send descending projections to the lateral lemniscal complex. The TSC projects to the auditory thalamic nucleus medialis through the tractus opticus lateralis. Physiological recordings from TSCv injection sites revealed a patchy distribution of best frequencies, while recordings in TSCd were characterized by broad frequency tuning. The tract tracing results revealed conserved anatomical patterns of ascending auditory connections in lizards and archosaurs.

## Graphical abstract

We used tract tracing and in vivo physiology to investigate auditory processing in the brainstem of the Tokay gecko. We found conserved patterns of connection in higher‐order auditory nuclei and an apparent lack of tonotopy at the level of the auditory midbrain.

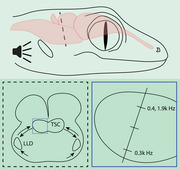

## 1 Introduction

Ambient sound conveys information about both the location and biological significance of acoustic sources. Correspondingly, auditory processing is characterized by circuits serving sound localization and recognition. In birds and mammals, these two processes are carried out by mainly independent but overlapping neural pathways (Grothe et al. 2004; Krützfeldt et al. 2010a). Less is known about the parallel processing of sound localization and recognition in other vertebrates (Carr and Code 2000; Woolley and Casseday 2004). We have therefore described the anatomical connections of a vocal lizard, the Tokay gecko (*Gekko gecko*). Many geckos produce loud, stereotypic calls, implying a long‐distance communication that requires both accurate localization and recognition of sound sources (Tang et al. 2001).

Among tetrapods, tympanic hearing has evolved multiple times in the lineages leading to modern amphibians, reptiles, and mammals (Clack 1997, 2015). Neural circuits for auditory processing have been studied extensively in model animals within these lineages such as frogs (Feng and Lin 1991; Horowitz et al. 2006; Walkowiak 1988), birds such as finches, chickens, and barn owls (Conlee and Parks 1986; Covey and Carr 2005; Krützfeldt et al. 2010a; Takahashi and Konishi 1988b), and mammals including bats, mice, and gerbils (Ito and Malmierca 2018). These animals have comparable ascending neural circuits (Carr and Christensen‐Dalsgaard 2016; Grothe et al. 2004; Walton et al. 2017), revealing a common octaval pattern of organization, which includes monaural and binaural nuclei in the medulla, convergence of multiple medullary projections in the auditory midbrain, and increased specificity of sound source localization (Carr and Edds‐Walton 2008; Grothe et al. 2004; Walton et al. 2017). These common features among divergent lineages are consistent with an ancestral “template” for auditory processing (Walton et al. 2017). These features are also consistent with an ancestral octavolateralis pattern exhibited by ascending lateral line, electrosensory, and eighth nerve systems (Fritzsch 1992; Grothe et al. 2004; Northcutt 1980).

Phylogenetic constraints on the hearing system may underlie the basic organization of the auditory pathways, while ecological adaptations should contribute to the diversification of auditory nuclei and connections. For example, all reptiles, including birds and lizards, have two cochlear nuclei, magnocellularis and angularis (Parks and Rubel 1978; Takahashi et al. 1984; Yan et al. 2010), but differently organized inner ears. Squamates have evolved specialized high‐best‐frequency regions of the inner ear, which are correlated with high‐ and low‐best‐frequency subdivisions within the nucleus angularis (NA) (Manley, Yates, et al. 1988; Szpir et al. 1990). No such divisions are found in birds, which have a tonotopically organized basilar papilla (Manley and Gleich 1992) and tonotopically organized cochlear nuclei. In the tetrapods studied to date—frogs, birds, and mammals—subdivisions of the nuclei of the auditory lemniscus and the torus semicircularis appear to have differentiated in parallel, with distinct patterns of innervation (Feng and Lin 1991; Oliver 2005; Schwartz 1992; Takahashi and Konishi 1988a, 1988b). The anatomy of these complexes remains largely unknown in squamate taxa (Foster and Hall 1978; Kennedy and Browner 1981). Investigation of gecko auditory nuclei and their connections should therefore shed light on the evolution of the vertebrate auditory system.

Vocal communication in lizards is mostly found in geckos. Male geckos make different calls toward males than those directed toward females, and vice versa for females (Frankenberg 1982; Hibbitts et al. 2007; Jono and Inui 2012). Individuals can form two types of call duets during the breeding seasons (Tang et al. 2001; Yu et al. 2011). Thus, this group may be a model for studies of the structure and connections of the higher order auditory pathways involved in sound localization and call recognition. In a previous study, we used tract‐tracing techniques to reveal the connections of the auditory brainstem in the gecko (Tang et al. 2012). Here, we investigate the afferent and efferent projections of the nuclei of the lateral lemniscus and torus in *G. gecko*, using neural tract tracing following electrophysiological recordings.

## 2 Methods and Materials

Adult Tokay geckos (*G. gecko*) of both sexes were used in the present study. Geckos from the 2012 study were wild caught in Southeast Asia, and the new geckos were wild caught in Fort Myers, FL (Swamp Creature Exotics, FL). All procedures were approved by the Animal Care and Use Committee of the University of Maryland, College Park. Data were obtained from cases described in our previous paper (Tang et al. 2012) and four new cases. All cases used similar tissue processing, data collection, and image preparation.

### 2.1 Anesthesia, Surgery, and Physiological Recordings

For the injection of tract tracers, geckos were pre‐anesthetized with a mixture of isoflurane and room air in a small chamber for about 10 min. Then, a small plastic tube connected with a vaporizer and oxygen cylinder was inserted loosely into the trachea to maintain anesthesia by providing isoflurane at 3% for surgery and 1% for recording. Experiments were conducted in a sound‐attenuating chamber with geckos maintained around 25°C using a heating blanket controlled by a feedback probe. The head was held in a constant position by gluing a stainless‐steel head‐post onto the prefrontal bone; a custom‐made sound system containing commercial miniature earphones and miniature microphones (EM3068, Knowles Electronics, Itasca, IL) was sealed to the ear entrance of both sides using Gold Velvet II materials (Gold Velvet, Oklahoma City, OK); and the sound system was calibrated. The midbrain was exposed through a dorsal craniotomy. If necessary, a small probe was inserted between the midbrain and cerebellum to access the caudal part of the torus. Physiological recordings were performed with 20‐MOhm tungsten microelectrodes, followed by recordings with a glass microelectrode containing tracer. The best frequency for each penetration was derived by measuring changes in spike rate in response to changing frequency (iso‐level response curves) from 100 to 5 kHz in 100‐ to 250‐Hz steps. Following physiological recordings, biotinylated dextran amine (BDA; 10%, 1:1 mix of 10,000 and 3000 MW; Invitrogen, Carlsbad, CA) or neurobiotin (4%, RRID:AB_2336606; Vector Laboratories, Burlingame, CA) was iontophoresed under an alternating positive current of 1–2.5 µA, with 7 s on and 7 s off, for 10–20 min. Geckos recovered for 1–4 days in their home cages before being euthanized. For perfusion, animals were injected with Euthasol (Virbac, West Lake, TX) i.m. at 7 mg/kg. After being euthanized, geckos were perfused. Brains were then removed, cryoprotected, and sectioned. Note that both dye spread and lack of single‐unit resolution constrain the interpretation of physiological data.

Sections were incubated in the avidin–biotin–peroxidase complex (ABC, Vector Laboratories), and tracer was visualized by the horseradish peroxidase substrate reagent SG kits (Vector Laboratories). For neurobiotin injections, a similar procedure was used, with sections processed using the ABC reagents. Standard Nissl protocols were used to counterstain sections. Labeled neurons, axons, and terminals and the contours of each nuclear group, were photographed and drawn using computer software (RRID:SCR_00177, Neurolucida; Microbrightfield, Colchester, VT) connected to a microscope (Olympus, BX60, New York, NY). Microphotographs were cropped, and their brightness and contrast were adjusted using Photoshop (Adobe, USA). All auditory nuclei were outlined, and labeled neurons and terminals were marked using the Neurolucida system.

### 2.2 Rapid Golgi

A modified Golgi–Cox technique was used on three geckos (Glaser and Van der Loos 1981; Ramón‐Moliner 1970; Tang et al. 2012). Animals were anesthetized with ketamine (20 mg/kg; Ketaject; Phoenix Pharmaceuticals, St. Joseph, MO), followed by a lethal dose of pentobarbital (20 mg/kg i.m.; Abbott Laboratories). Brains were removed and placed in Golgi fixative containing potassium dichromate, mercuric chloride, and potassium chromate (Rapid Golgi Kit; FD NeuroTechnologies, Ellicott City, MD), then sectioned in the transverse plane.

### 2.3 Data Analysis

Injection site locations were determined by combining observations of label spread, recording depth, and physical location. The presence of densely labeled neurons and neuropil was used to confirm the injection sites. Fortunately, most auditory structures, except the SOd, were located near either the dorsal or the ventral surface of the brain, assisting in localization. Support for injection sites being confined to a particular structure was that both small and large injections yielded qualitatively similar anterograde and retrograde results. Labeled neurons, axons, and terminals and the contours of each nuclear group were photographed and drawn using computer software (Neurolucida) connected to a microscope (Olympus BX60). Sections with fluorescent label were photographed with a Zeiss 710 confocal microscope. Labeled terminals were measured by manually selecting and circling the targeted region in ImageJ (NIH open source), which calculated their area. Stained axons were partially reconstructed to identify tracts, and all labeled neurons and terminals were counted manually using the Neurolucida system (Table 1).

**TABLE 1:** Neurons and terminals labeled after tracer injection to a specific nucleus.

Physiology	Animal#	Injection site^a^	Label (+cell bodies, *terminals, ^axons only)															
			Ipsilateral								Contralateral							
			TSCv	TSCd	TSS	LLD	SOv	SOd	NA	NL	TSCv	TSCd	TSS	LLD	SOv	SOd	NA	NL
	42	TSCd	*			*	*				*			*	*			
Yes	78	TSCv		+	*	+*	+*	+			+*	+		+*	+*	+		
Yes	80	TS				+*	+*	*			+*			+*	+*	+	+	+
	107	NM/NL				*	*							*	*			
Yes	116	TSC				+*	+*											
	117	LLD	+	+			+							+	+			
Yes	119	TSC rostral				+	+*							+	+	+	+	
Yes	137	TSCv		+	*	+++*	+++*	+			*	+				+		
	143	NA				+	+*	?						*	*	?		
	149	NA				*	+*	+*			*			*	*	*		
Yes	153	TSCv		+	*	+*	+					+				+		
	155	NM/NL				*	*							*	*			
Yes	156	TSCv			*	+*	+				+*						+	
	157	NA + NL + VeO	+*			+*	+*	+*			+*			+*	*	*		
	163	SOd				*	*		++	++	*			*	*			
	166	SOv				^												
	167	SOv				^												

*Note:* Number of symbols (+, *) denotes strength of label, that is, numbers of terminals and cell bodies.

^a^Cases 42–119 were labeled using BDA, and cases 137–167 were labeled using neurobiotin.

## 3 Results

We had previously shown that the auditory nerve projects to NA and the nucleus magnocellularis (NM) and that NM projects bilaterally to the nucleus laminaris (NL). These nuclei receive descending connections from the superior olivary nuclei (SO) (Tang et al. 2012). Here, we describe the ascending projections of NA, NL, and SO to the lemniscal nuclei and auditory midbrain and reveal connections of the auditory midbrain, including descending connections to the lemniscal nuclei and ascending projections to the thalamus.

### 3.1 Organization of Auditory Nuclei

**FIGURE 1:**
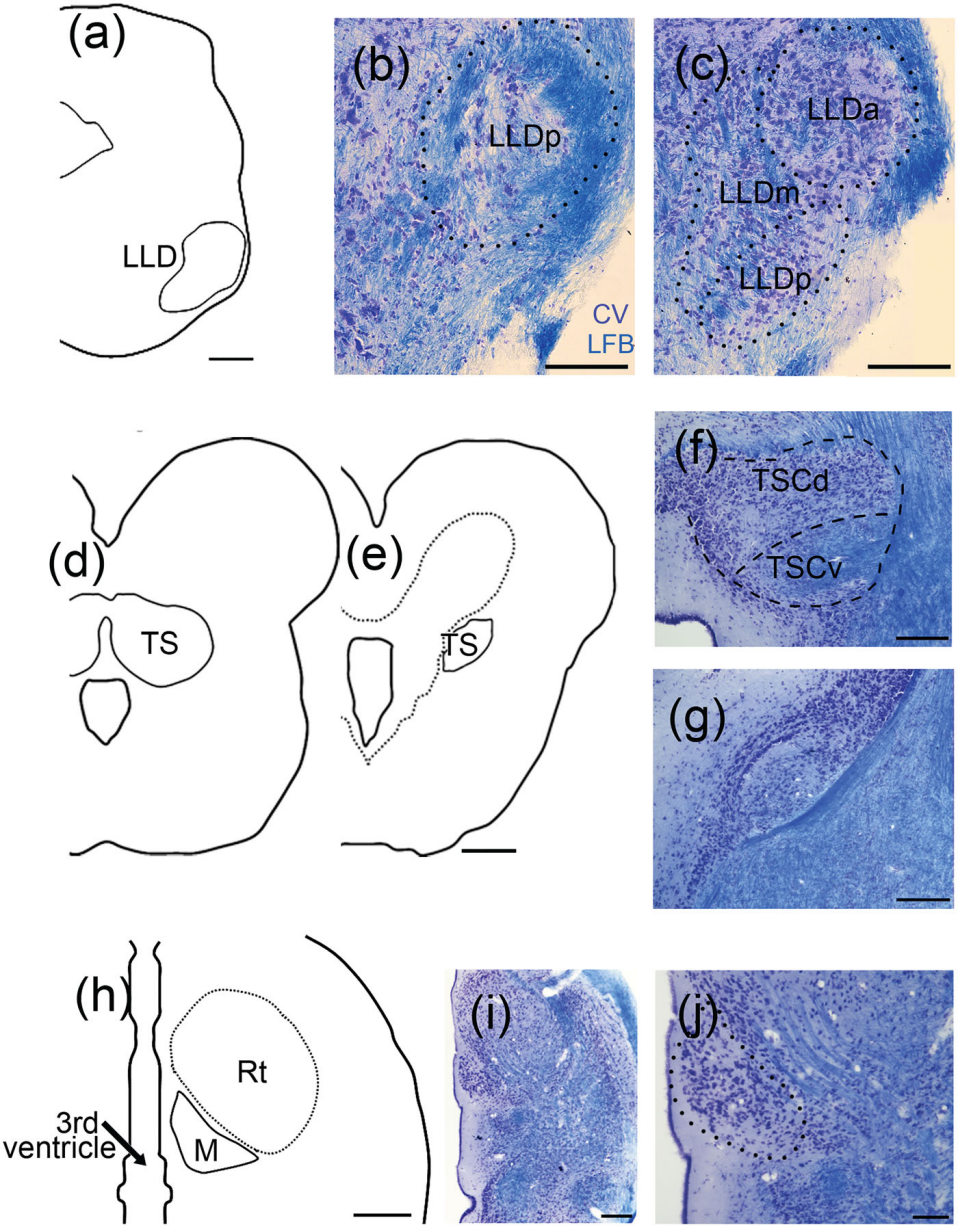
Drawings (left) and photomicrographs (right) of a series of transverse sections from caudal to rostral, stained with cresyl violet (CV) and Luxol fast blue (LFB). For all drawings and photomicrographs, medial is to the left, right is lateral, top is dorsal, and bottom is ventral in orientation. (a) The dorsal nucleus of the lateral lemniscus (LLD) is located ventral in the rostral medulla and has three subdivisions. (b) Posterior LLD (LLDp; dotted line) is most caudal in the lemniscal complex and is bounded laterally by myelinated lemniscal axons. (c) More rostrally, LLD has three divisions (black dotted lines): anterior (LLDa), medial (LLDm), and posterior (LLDp). Note the lemniscal axons lateral to LLDa. (d) The central nucleus of the torus semicircularis (TSC) appears fused at the midline in the caudal midbrain. (e) Rostrally, TSC extends in a “wing” below the tectal ventricle. (f) The two subdivisions (black dotted lines) of the central nucleus: dorsal (TSCd) and ventral (TSCv). These are outlined by lemniscal axons, which enter TSC ventrally and laterally. (g) TSC subdivisions are difficult to distinguish in rostral portions. (h) The nucleus medialis (black dotted line; M) is located just lateral to the third ventricle, below nucleus rotundus (Rt). (i, j) The nucleus medialis contains large oval neurons. Scale bar for drawings: 500 µm (a, d, and h); 50 µm (b and c); 200 µm (f, g, and i); and 100 µm (j).

Sections through the brainstem, midbrain, and thalamus were counterstained with cresyl violet, and fiber tracts were stained with fast blue (Figure 1), allowing us to identify three auditory groups between the rostral brainstem and thalamus. These were the dorsal nuclei of the lateral lemniscus (LLD), the central nucleus of the torus semicircularis (TSC), and the nucleus medialis of the thalamus (M).

The nuclei of the lateral lemniscus are in the lateral and rostral hindbrain, between the olivary nuclei and TS, and form a complex ventral to TS (Figure 1a). The complex also includes a nucleus caudal and ventral to LLD, the ventral superior olive (SOv) (Tang et al. 2012; Yan et al. 2010). LLD is surrounded by the thick axons of the spinal lemniscus and by lateral lemniscus fibers, with the greatest density of axons at the lateral edge of the brainstem (Figure 1b,c). LLD has three subdivisions, based on position, neuronal shape, and size. A round anterior division contains the largest neurons (LLDa) and is located both anterior and dorsolateral to the posterior division (LLDp), which has smaller neurons (Figure 1c). These subdivisions had previously been identified in immunohistochemical material using antibodies against calcium‐binding proteins and glutamic acid decarboxylase (Yan et al. 2010). A third subdivision, identified as a small group of neurons medial to LLDa and LLDp, did not show either a clear boundary or neuronal cluster but could be recognized in tract‐tracing experiments (LLDm, see below) (Figures 1c and 8c).

Kennedy and Browner (1981) divided the torus semicircularis of the Tokay gecko into central, laminar, and superficial nuclei. The central nucleus (TSC) forms the major component of TS, and the laminar nucleus forms a cap medial and dorsal to TSC at rostral levels. Contrary to Kennedy and Browner (1981), we did not observe a clear caudal extension of the laminar nucleus, which was described as encircling the caudal TSC. The superficial nucleus is located dorsal and dorsolateral to the central nucleus and is separated from TSC by white matter tracts. Neither previous studies nor our data support an auditory role for the laminar or the superficial nuclei (Díaz et al. 2000; Foster and Hall 1978; Kennedy 1975), so we have focused on TSC in the present study.

In the Tokay gecko, TSC is large and occupies most of the caudal, dorsal midbrain (Figure 1d). Caudally, it is fused at the midline and, more rostrally, bifurcates to form an elliptical profile beneath the tectal ventricle (Figure 1d), where it decreases in size toward the rostral midbrain (Figure 1e). We have divided TSC into two subregions, dorsal (TSCd) and ventral (TSCv) (Figure 1g). We had previously used the spatial pattern of calcium‐binding protein expression to divide the gecko TSC into three subregions—lateral, ventral, and dorsal (Yan et al. 2010). In the present study, we have combined the lateral and ventral divisions into a ventral division and will show here that ascending brainstem projections converge on this ventral division.

**FIGURE 2:**
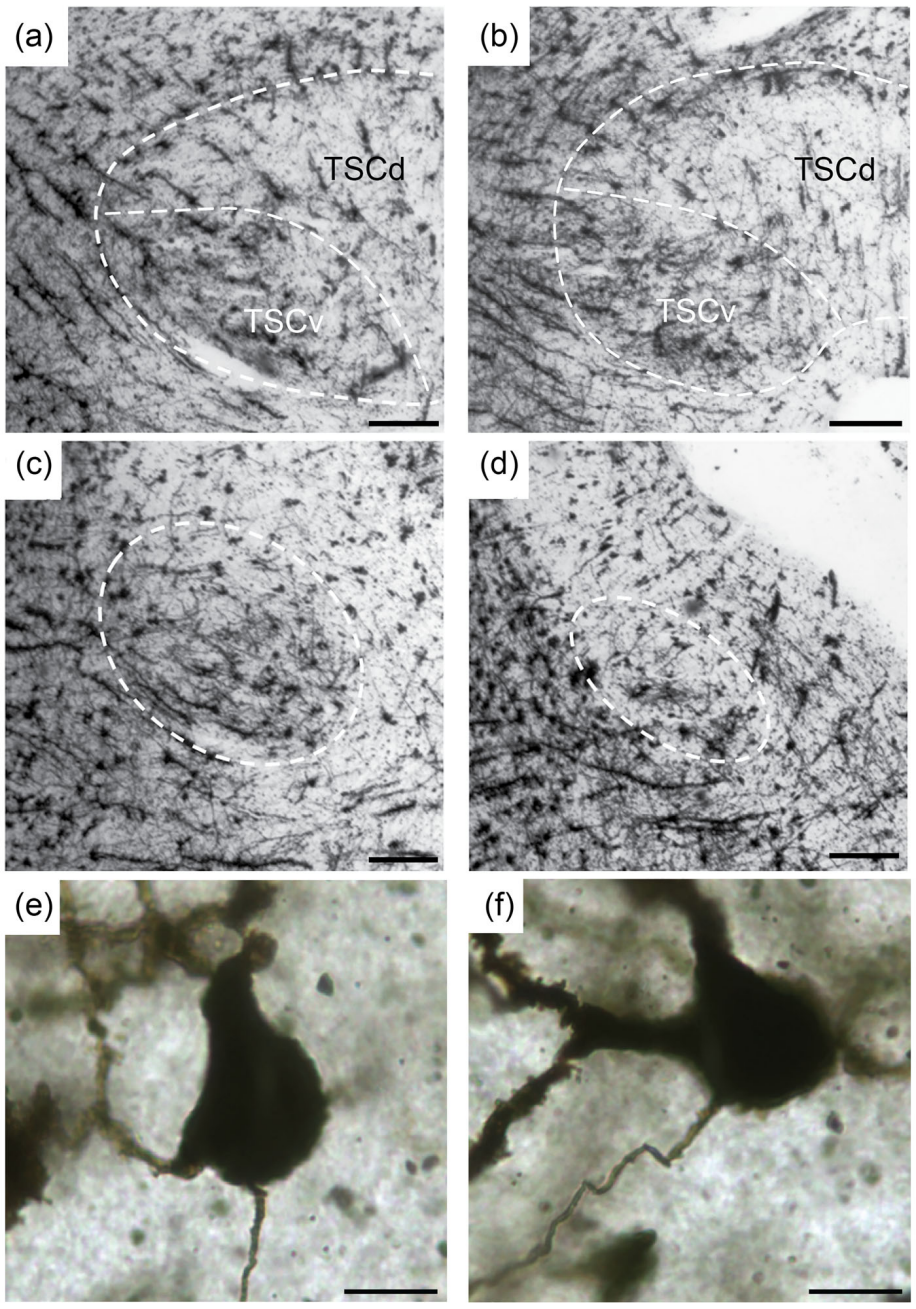
TSC is separated into dorsal and ventral subdivisions in Golgi‐stained sections. (a) The caudal TSC reveals an even distribution of neurons and fibers. The border between TSCd and TSCv is shown by a dashed line. (b) More rostrally, TSC is characterized by neuronal clusters in TSCv. Section located posterior to Figure 1f. (c) TSC (dashed lines) divides to form a wing below the superficial nucleus of TS; note Golgi‐stained neurons in the central nucleus. (d) The most rostral portion of TSC (dashed lines) forms an oval in a transverse section (see Figure 1g). (e) Cell body and axon characterize a triangular cell in TSCv (Kennedy and Browner 1981). (f) Cell body, axon and primary spiny dendrites of a large spherical neuron in TSCv (Kennedy and Browner 1981). Scale bar = 200 µm (a–d) and 10 µm (e–f).

Caudally, both ventral and dorsal divisions adjoin their contralateral homologs at the midline. The two subdivisions could be differentiated in Golgi‐stained material, since the neuropil of the ventral division is distinct from surrounding areas (Figure 2). In Golgi material, the ventral and dorsal subdivisions are more differentiated in the caudal torus (Figure 2a,b) and become less apparent in rostral sections where TSC is situated beneath the tectal ventricle (Figure 2c,d). Consistent with results from Kennedy and Browner (1981), fusiform neurons were numerous and distributed equally among the two subregions. Spherical cells were also found throughout the central nucleus but were less common in the Golgi material. There were fewer impregnated neurons in the dorsal subdivision than in the rest of the central nucleus.

### 3.2 Ascending Projections From the Brainstem Cochlear Nuclei

Tracer injections into NA and/or NM/NL labeled axons in the lateral lemniscus and terminals in the lemniscal nuclei and TSC, almost exclusively in the ventral division (TSCv). Projections to the lemniscal nuclei were bilateral, while projections to TSC were skewed contralateral (Figure 5a).

**FIGURE 3:**
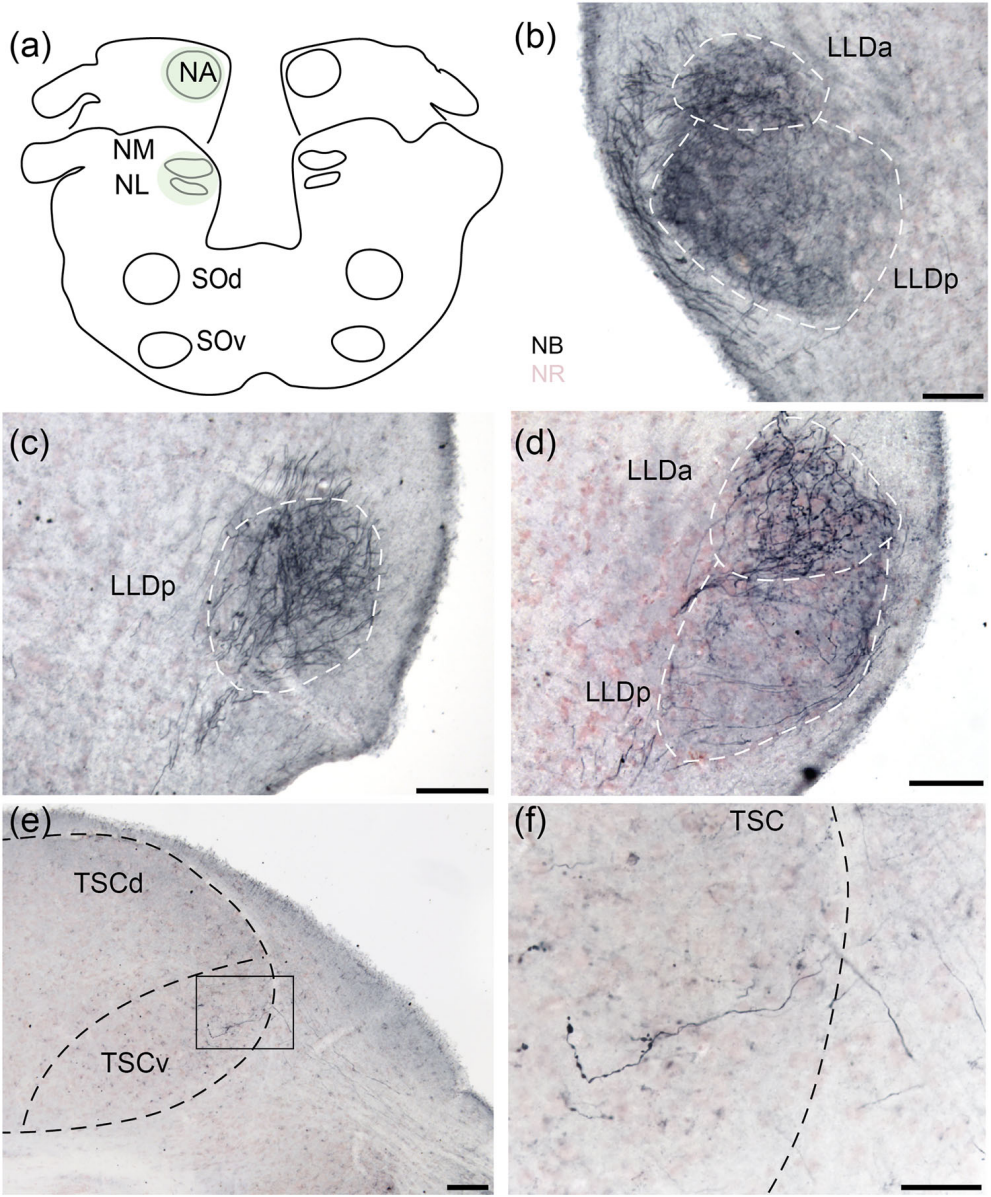
The cochlear nucleus complex projects to the lemniscal nuclei and torus semicircularis. (a) Schematic drawings of the rostrocaudal extent of the injection site in GG157 (green overlay). The injection site includes NM, NL, and NA. (b) Labeled fibers and terminals in the ipsilateral LLDa and LLDp (white dashed lines). NB, neurobiotin; NR, neutral red for panels (b–f). (c) Labeled fibers and terminals in the contralateral LLDp (white dashed lines). (d) Labeled fibers and terminals in the contralateral LLDa and LLDp (white dashed lines). At this level, the labeled terminal field is densest in LLDa. (e) Labeled fibers and terminals in the contralateral TSCv (black dashed lines). (f) High‐magnification image showing details of the labeled fibers from the box in (e). Scale bar = 500 µm (a), 100 µm (b–e), and 50 µm for (f).

A large injection into the dorsomedial acoustic tubercle that included NM, NL, and NA (GG157; Figures 3a and 5a) yielded labeled lemniscal fibers bilaterally. As reported in Tang et al. (2012), these injections yielded retrogradely labeled neurons in the ipsilateral SOd and SOv, and terminal fields in SOd and SOv bilaterally. Most ascending fibers travelled lateral to SOv, close to the brain surface, and formed dense terminal fields in the ipsilateral LLDp and LLDa (Figure 3b and 5a). The contralateral lemniscal fibers were located more medially in the lateral lemniscus and also terminated in LLDp (Figure 3c) and LLDa (Figure 3d). The ascending axons continued to traverse rostrally and followed a fiber tract close to the lateral edge of the magnocellular isthmic nucleus to terminate in the lateral part of TSCv (Figures 3e,f and 5a). Similar degrees of innervation were seen between the ipsilateral and contralateral LLD. More densely labeled axons and terminals were found in the contralateral TSCv compared to the ipsilateral. A small injection into NA alone (GG149) yielded a similar pattern of labeled fibers and terminals to GG157. Small injections (GG107, GG155) that labeled NM/NL but excluded NA resulted in a few fibers and terminals stained in LLDp bilaterally. Our companion report showed that NM projects bilaterally to NL, with an ipsilateral projection to the dorsal NL neuropil and the contralateral projection across the midline to the ventral dendrites of NL neurons (Tang et al. 2012). Since NL is small and close to NM, an injection into NL extended to the whole NM/NL region (Figure 3a).

A large injection in TSC (GG80), along with two smaller injections, one in the rostral TSC (GG119) and one in the caudal TSCv (GG156), yielded retrogradely labeled cells in the contralateral NA. The large injection (GG80) also labeled contralateral NL neurons. This corroborates the results from anterograde labeling, suggesting that NA/NL recipient areas are likely distributed along the rostrocaudal axis of TSC and primarily located in the lateral TSCv at caudal levels. Small injections restricted to TSCd did not label cells in the brainstem cochlear nuclei. Retrograde label supported our observation that projections from the acoustic tubercle to TSC were primarily contralateral.

### 3.3 Ascending and Reciprocal Connections From the Superior Olivary Nuclei

Geckos have two superior olivary nuclei, one dorsal and one ventral (Figures 3a and 5) (Tang et al. 2012; Yan et al. 2010). Both nuclei form rostrocaudally directed cell columns, with the ventral nucleus (SOv) originating just caudal to LLD and extending to the level of the first‐order nuclei (Yan et al. 2010). SOd is more restricted in range. SOd and SOv both project to LLD and TSC.

**FIGURE 4:**
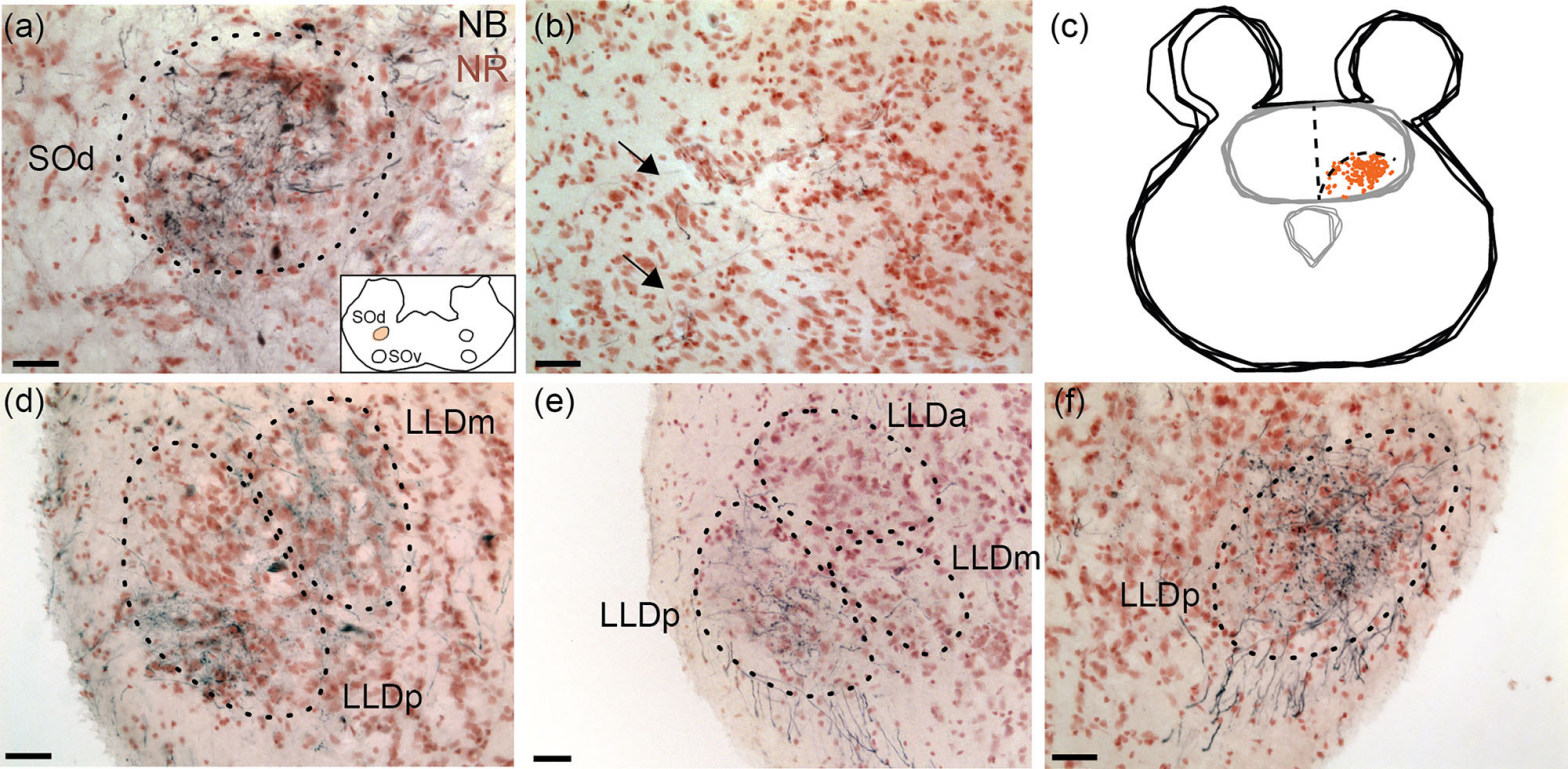
The dorsal superior olive (SOd) forms connections with TSC and LLD (GG163). (a) Injection site in SOd (dotted lines). NB, neurobiotin; NR, neutral red for panels (a), (b), and (d–f). Inset: Schematic of injection site, orange overlay. (b) The contralateral part of TSCv contained fibers and terminals following SOd injection (arrows). (c) Neurolucida reconstruction of labeled terminals in TSCv following neurobiotin injection into SOd. Each terminal is marked to aid visualization. (d) Fibers and neurons in the ipsilateral LLDm and LLDp (dotted lines) following dye injection in SOd. (e) Comparatively, fewer fibers and neurons were labeled in the ipsilateral LLDa (dotted lines). (f) Labeled fibers and terminals in the contralateral LLDp (dotted lines). Scale bar = 50 µm for all.

**FIGURE 5:**
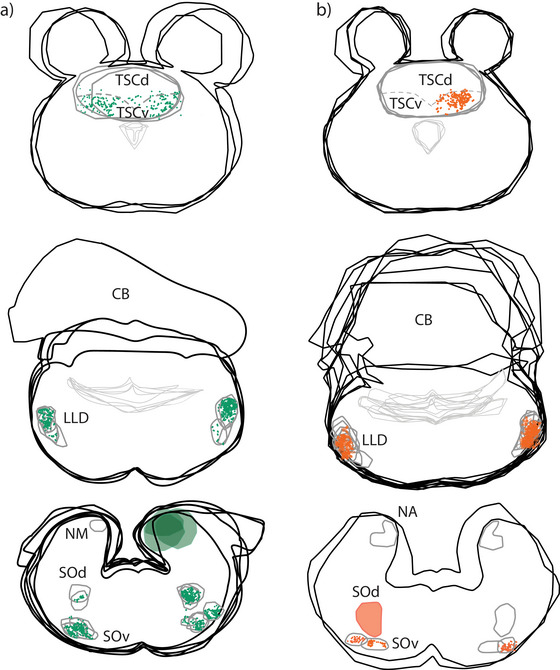
Neurolucida reconstruction of labeled cells and terminals after two injections into the acoustic tubercle and SOd. Three levels are shown with superimposed transverse sections of the brainstem nuclei and more rostral lemniscal nuclei and TSC. Green and orange fill matches Figure 13. (a) Large injection into the acoustic tubercle (GG 157, green fill) labeled NM, NL, and NL. The injection yielded retrogradely labeled neurons in the contralateral NM (not shown) and bilateral terminals in SOd, SOv, LLD, and TSCv (green symbols). (b) Injection into SOd (GG163, orange fill) with terminals in both the ipsi‐ and contralateral SOv and LLD (orange symbols). The injection yielded retrogradely labeled neurons in the contralateral NA (not shown). Note the sparse contralateral projections to TSCv.

An injection in SOd (GG163; Figure 4a, injection site) revealed axons and terminals in the contralateral TSCv (Figures 4b and 5b). Similar connections were found following injections in TSC, with retrogradely labeled cells in SOd being mainly contralateral (see Tang et al. 2012). In addition, SOd injections yielded labeled fibers and terminals in LLDp and LLDm on both sides, with LLDp containing more labeled axons and terminals than LLDm (Figure 4d–f). Projections to LLDa were sparse.

**FIGURE 6:**
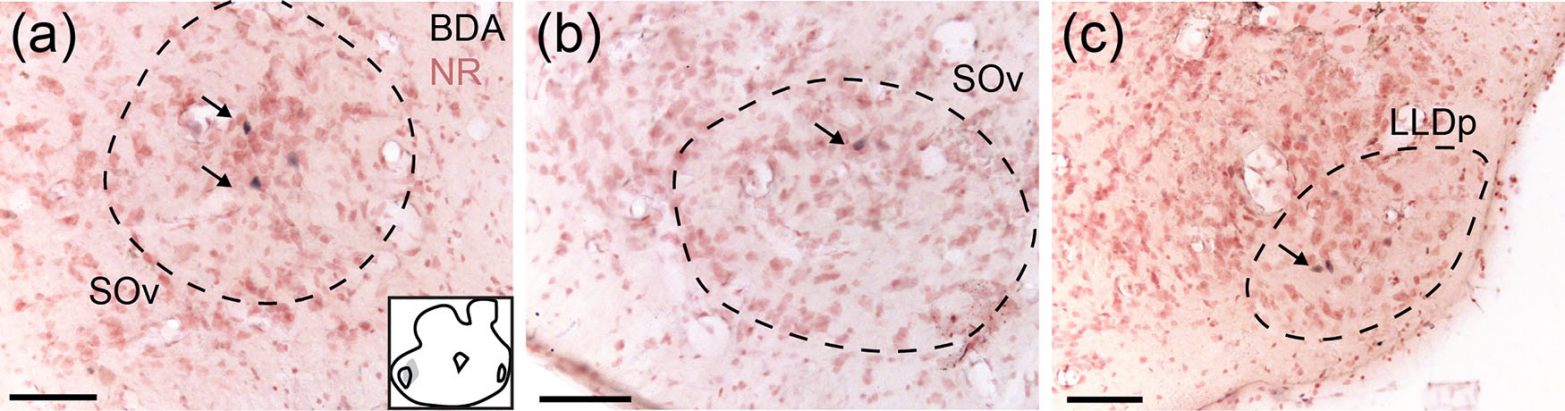
LLD receives connections from its contralateral homolog and SOv (GG117). (a) Retrogradely labeled neurons (arrows) in the contralateral SOv following a large injection in LLD. NB, neurobiotin; NR, neutral red for all panels. Inset: Schematic of the injection site, gray overlay. (b) Retrogradely labeled neurons (arrow) in the ipsilateral SOv after the same injection. (c) Retrogradely labeled neurons (arrow) in the contralateral LLDp. Scale bar = 100 µm for all.

Injections in SOv (GG166, GG167) showed limited dye transport and yielded only a few fibers in the ipsilateral LLD. Nevertheless, a large injection into LLD retrogradely labeled cells in SOv bilaterally (GG117), suggesting connections between SOv and LLD (Figure 6a,b). Large injections in TSC and a smaller injection in TSCv retrogradely labeled cells in SOv, with cells mainly on the ipsilateral side. All TSC injections produced retrogradely labeled cells in SOv, whereas only some injections labeled cells in SOd. In the cases where both SOv and SOd were labeled, there were more labeled cells in SOv than SOd (see Tang et al. 2012).

**FIGURE 7:**
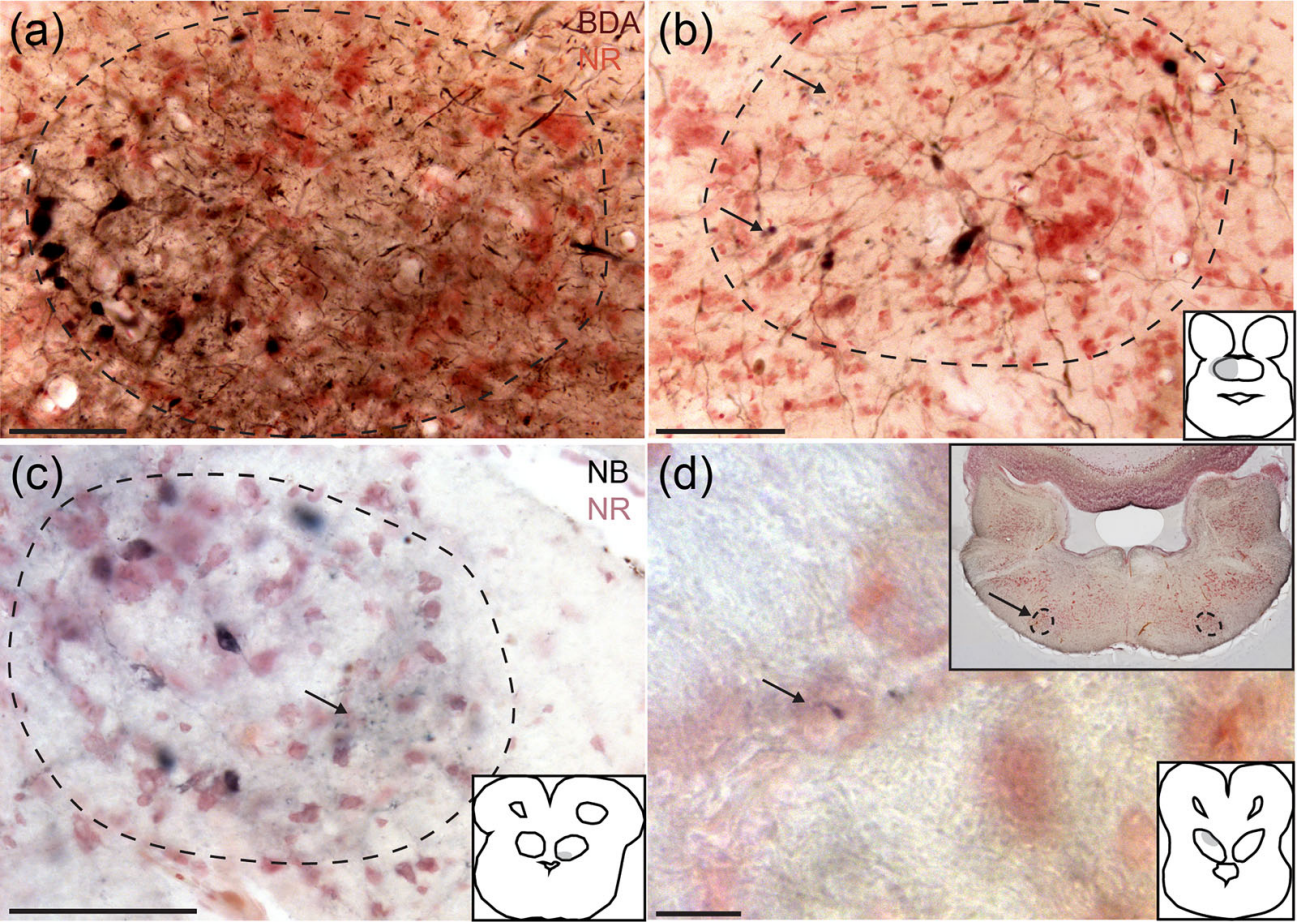
SOv receives descending projections from TSC. (a) Retrogradely labeled neurons and terminal field in the ipsilateral SOv following a large injection in the TSC (GG80). (b) Retrogradely labeled neurons and terminals (arrows) in the contralateral SOv. BDA, biotinylated dextran amines; NR, neutral red for panels (a), (b), and (d). Inset: Schematic of the injection site for (a) and (b), gray overlay. (c) Labeled terminals (arrow) in the ipsilateral SOv following a small injection in TSCv (GG137). NB, neurobiotin; NR, neutral red. Inset: Schematic of the injection site, gray overlay. (d) Labeled terminals (arrow, Nomarski optics) in the ipsilateral SOv following a small injection in TSCd (GG42). Inset top: Location of the labeled terminals in a transverse section. Inset bottom: Schematic of the injection site, gray overlay. Scale bar = 100 µm (a–c) and 10 µm (d).

Injections in TSC also labeled terminals in SOv, mainly on the ipsilateral side, suggesting a reciprocal connection (Figure 7). Notably, a small injection in TSCd (GG42) produced labeled terminals in SOv (Figure 7d). No descending projections were found from TSC to SOd.

### 3.4 Ascending and Reciprocal Projections From LLD

**FIGURE 8:**
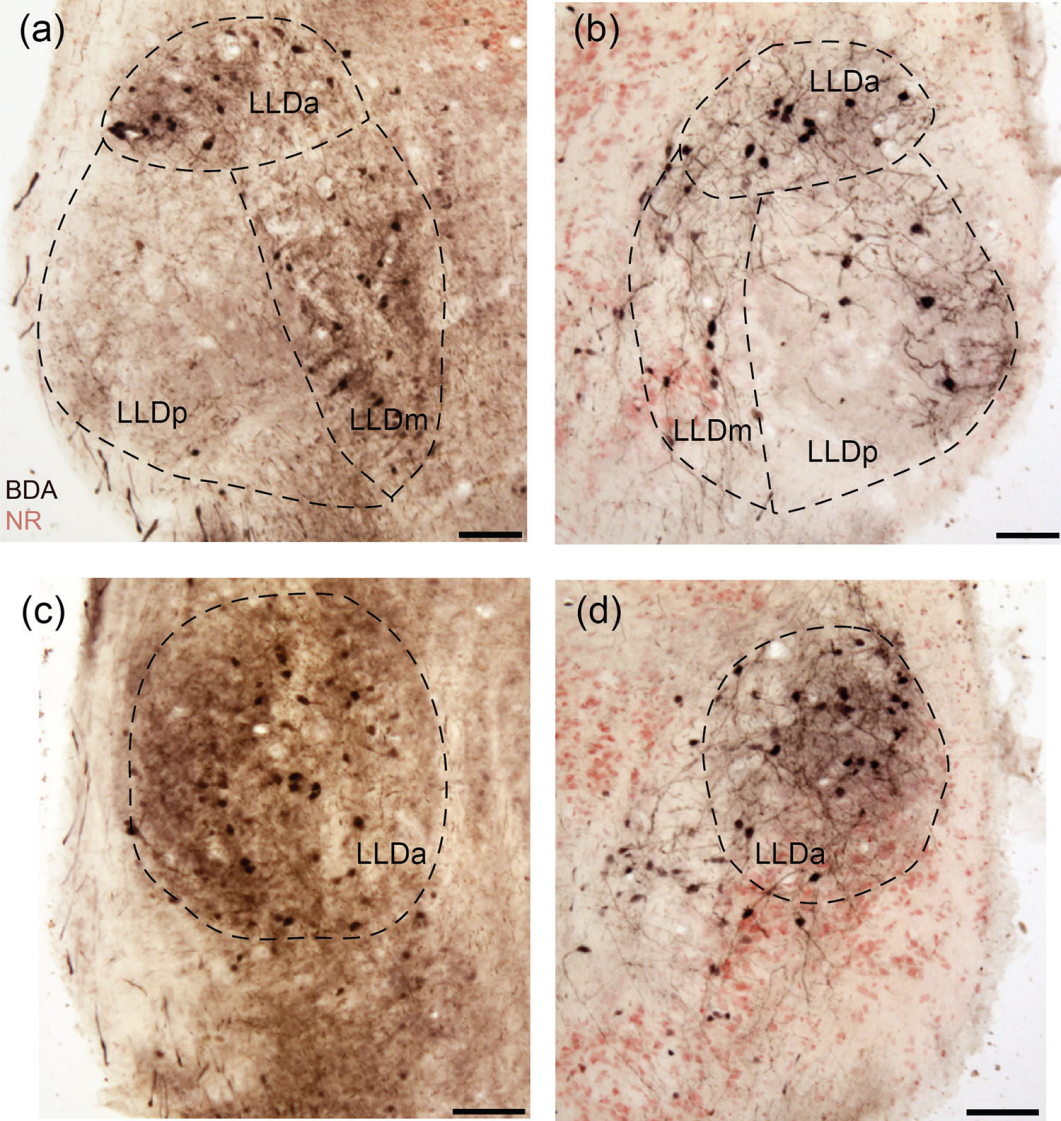
Reciprocal connections between the LLD and torus semicircularis (TSC) after a large BDA injection (GG80). (a) Neurons and terminals labeled in the ipsilateral LLDa and LLDm (dotted lines) in a caudal section. Limited label was seen in LLDp (dotted lines). Injection site can be found in Figure 7b. (b) Neurons and terminals labeled in the contralateral LLD (dotted lines) in a caudal section. (c) Neurons and terminals labeled in the ipsilateral LLDa (dotted lines) in a rostral section. (d) Neurons and terminals labeled in the contralateral LLDa (dotted lines) in a rostral section. Scale bars = 100 µm for all.

The TSC receives projections from LLD. A large injection in TSC (GG80) retrogradely labeled cell bodies in all subdivisions of LLD bilaterally (Figure 8). A smaller injection in the rostral TSC (GG119) produced similar results. Three other small injections in TSCv (GG78, GG137, GG153) only labeled cells in the ipsilateral LLD, with very few fibers discernible on the contralateral side. An injection localized to the caudalmost tip of TSC, which labeled many NA cell bodies, did not retrogradely label cells in LLD. Additionally, a large injection in LLD (GG117) labeled cells in its contralateral homolog (Figure 6c).

TSC and LLD are reciprocally connected. An injection in LLD (GG117) anterogradely labeled cells in TSC. In transverse sections, cell bodies were found throughout TSC, including TSCd, an area that received almost no ascending input from the cochlear nuclei. More cell bodies were labeled in the rostral TSC than in the caudal TSC. Conversely, labeled terminals could be seen in LLD following injections into TSCv and TSCd. Similar to the ascending projections from LLD to TSC, the descending connection is also more prominent on the ipsilateral side (Figure 8). For both sides, the connection strength between TS and each subnucleus of LLD appeared strong between both LLDa and LLDm, and weaker between TS and LLDp (Figure 8).

### 3.5 Additional Projections From TSC

**FIGURE 9:**
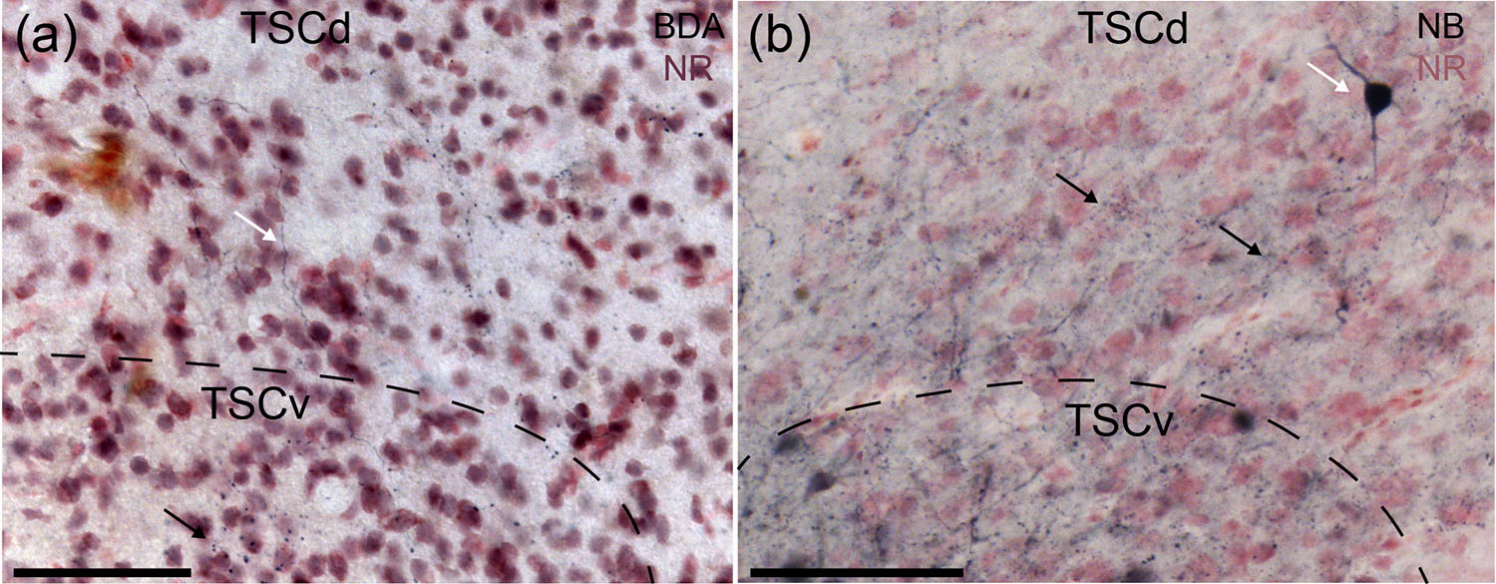
TSCv and TSCd form reciprocal connections. (a) Labeled axons (white arrow) course ventrally to terminate in TSCv (black arrow) following a small injection in TSCd (GG42). BDA, biotinylated dextran amines; NR, neutral red. Injection site can be found in Figure 7d. (b) Labeled neurons (white arrow) and terminals (black arrows) in TSCd following a small injection in TSCv (GG137). NB, neurobiotin; NR, neutral red. Injection site can be found in Figure 7c. Scale bar = 100 µm for all.

Ascending projections from the acoustic tubercle and SOd terminated almost exclusively in TSCv. TSCv also forms reciprocal connections with TSCd (Figure 9). In a small injection in the rostral part of TSCd (GG42), labeled fibers coursed in ventral, caudal, and orthogonal directions to terminate in TSCv bilaterally (Figure 9a). Conversely, injections restricted to TSCv yielded labeled cells and terminals in TSCd (Figure 9b). All our TSC injections yielded labeled terminals in the ipsilateral superficial nucleus of the torus semicircularis (TSS). While it is possible that this could have been a result of dye spread in some cases, in one injection, we only labeled the medial portion of TSCv (GG137) and still observed terminals in the ipsilateral TSS, supporting the presence of a projection from TSC to TSS.

**FIGURE 10:**
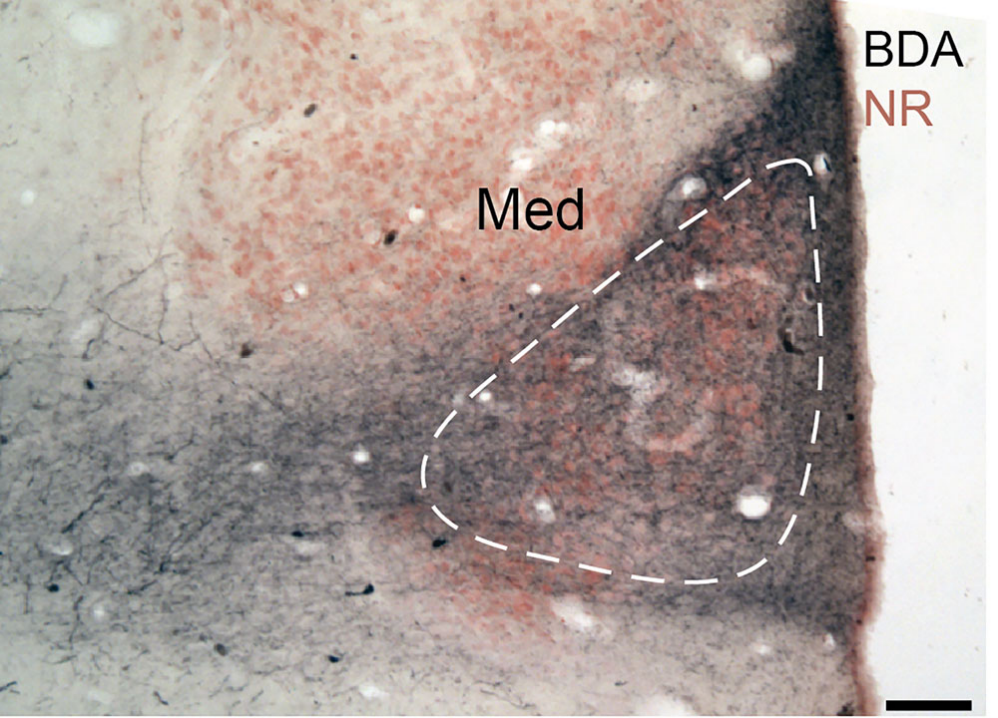
TSC projects to the nucleus medialis (Med). Labeled fibers and terminals in the contralateral nucleus medialis (white dashed lines) following injection in TSC (GG80). Scale bar = 100 µm. BDA, biotinylated dextran amines; NR, neutral red. Injection site can be found in Figure 7b.

In the diencephalon, TSC projected to the ipsilateral nucleus medialis of the thalamus. The nucleus medialis formed a triangular nucleus ventromedial to the nucleus rotundus against the third ventricle (Figure 1h–j). A large injection in TSC (GG80) yielded many fibers and terminals that filled the nucleus medialis (Figure 10), while a smaller injection (GG78) showed a dense terminal field in the ipsilateral nucleus medialis. In the caudal thalamus, some stained axons were found in clusters at the tractus opticus lateralis located laterally beneath the rostral tectum.

### 3.6 Physiological Organization of TSC

**FIGURE 11:**
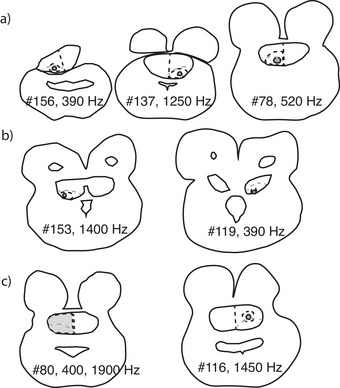
Injection sites in TSC and their best frequencies (BF). (a) The centers of each injection site in caudal and central TSCv. Case number from Table 1 and BF (Hz) shown adjacent to each contour, with the best frequency of the recording site. Gray dashed lines mark the border between TSCd and TSCv. (b) The center of two injection sites in the central and rostral TSCv. Case number from Table 1 and BF (Hz) shown adjacent to each contour, with the best frequency of the recording site. Gray dashed lines mark the border between TSCd and TSCv. (c) The center of two injection sites in the central and rostral TSCd. Case number from Table 1 and BF (Hz) shown adjacent to each contour, with the best frequencies of the recording site. Gray dashed lines mark the border between TSCd and TSCv.

**FIGURE 12:**
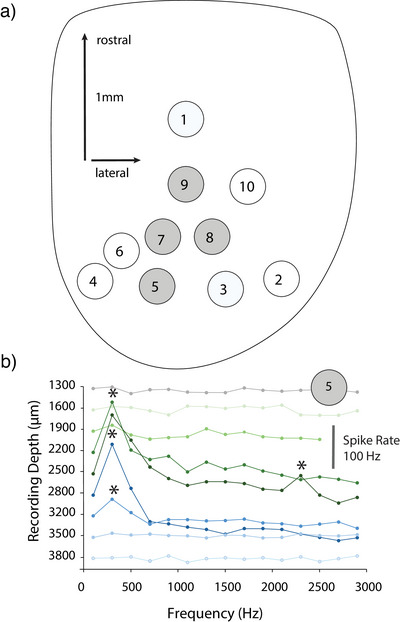
Recordings of best frequency in TSC. (a) Dorsal view of midbrain (optic lobe) in the experiment used to map frequency tuning in TSC. Gray numbered electrode penetrations contained auditory responses. (b) Penetration 5 plots responses for tonal stimuli for each recording depth. Spike rate indicated by vertical bar; *, responses significantly above baseline.

The anatomical observations presented here were supported by electrophysiological mapping of the injection sites (Figure 11). The injections described above were combined with multiunit recordings from dye‐filled electrodes in the torus in seven geckos, and results from these experiments will be reported briefly here to address the organization of TSC. Neuronal responses to airborne sound differed between the dorsal and ventral portions of the torus. Prominent responses to tonal stimuli were mainly found in TSCv, consistent with this region receiving ascending input from the acoustic tubercle, superior olivary, and lemniscal nuclei (Figures 11 and 12). The TSCv region is also delineated by parvalbumin immunoreactive cell bodies and ascending calretinin‐positive axons and terminals (Yan et al. 2010).

Recordings from dye‐filled electrodes revealed responses with best frequencies of 300–3000 Hz from TSCv (Figure 11a,b). One recording site in the medial TSCv (GG137) displayed a systematic tonotopic change with depth, with a shift in best frequency of 200–1200 Hz over 1 mm of recording depth. Other recordings from TSCv revealed a patchy distribution of best frequencies, with best frequencies of injection sites ranging from 390 Hz (GG156) to 1400 Hz (GG153) (Figure 11a). There was a similarly patchy distribution of best frequencies between caudal and rostral TSCv—that is, the three rostral TSCv injection sites ranged in best frequency from 390 to 1450 Hz (Figure 11b). With the exception of case GG137, we observed no systematic changes in best frequency. It is possible that future studies may divide the TSCv into regions that receive input from different brainstem sites.

In TSCd, recordings (GG80, GG116) typically displayed broad or multipeaked tuning curves. GG80 recordings showed best frequencies around 400 and 1900 Hz (Figure 11c) throughout the dorsoventral extent of TSCd, while the GG116 recording site had a low frequency peak at 500 Hz and a second peak at 1400 Hz. Other recordings in TSCd were characterized by broad tuning curves.

**FIGURE 13:**
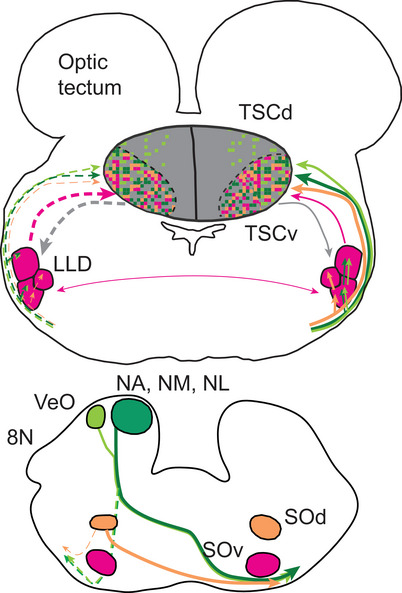
Schematic of the connections to LLD and TSC. Dashed lines indicate ipsilateral connections, and the width of the line estimates connection strengths. Projection from cochlear nuclei (NA and NM/NL) in dark green, projections from VeO in light green, projections from SOd in orange, ascending projections from LLD and SOv in pink, and descending projections from TSC in gray. In TSCv, it is not clear whether ascending projections from NA/NL, SO, and LLD and descending projections were separated into microdomains or intermixed.

To characterize the varied tuning observed at the sites of dye injection, we carried out a systematic review of frequency responses in TSC of a single gecko (Figure 12). Best frequencies were measured using binaural stimuli, delivered at 0 µs ITD and 75 dB at 100‐µm intervals in a series of 10 dorsoventral penetrations through the midbrain. After passing through the optic tectum, auditory responses were observed in four of the 10 penetrations in the caudomedial midbrain (gray circles, Figure 12a), corresponding to the TSC locations in Figure 1d,e. Multiunit auditory responses were first encountered at depths, measured from the surface of the optic tectum, between 1300 and 2200 µm. Initially, weak, broadly tuned responses were encountered, consistent with other recordings in TSCd. These were followed in all four auditory response penetrations (gray circles) by strong responses to stimuli around 300–400 Hz at a depth of about 2 mm before more ventral recordings yielded no further responses to auditory stimuli. The strong responses in the ventral third of TSC were consistent with the termination of ascending projections from hindbrain cochlear nuclei to TSCv (Figures 5 and 13).

### 3.7 Summary of Auditory Connections From Lemniscal and Olivary Nuclei

We compiled data from 20 animals to generate the summary of connections in Figure 13 and Table 1. NL and NA project to LLD bilaterally. NA projects to both LLDa and LLDp, and NL projects to LLDp. Injections that separately labeled NA and NL were small, and their projections were weak, so the difference between the projections from NA and NL is not definitive. NL and NA project to TSCv, mainly on the contralateral side. In a separate study, we found that the hindbrain saccular nucleus (VeO) terminated more dorsally in TSC (Han and Carr 2024). The termination pattern from the nuclei in the acoustic tubercle (NA/NL, VeO) mirrored the distribution of calretinin‐positive fibers in TSC, with VeO projections located in the caudalmost TSC and a small area in TSCd. Projections from NA/NL terminated in the caudalmost TSC and extended rostrally in TSCv.

Both SOd and SOv projected to LLD bilaterally. SOd projected to LLDp and LLDm, and projections from SOd to TSC were mainly contralateral (Figure 11b). Projections from SOv were mainly ipsilateral. Ascending and descending connections between LLD and TSC were mainly ipsilateral. The strength of this connection was strong in LLDa and LLDm, but weak in LLDp. There were also descending projections from TSC to the ipsilateral SOv. In the areas of TSC where subdivisions are discernible, the ascending projections from hindbrain cochlear nuclei are primarily localized to TSCv. Tracing results and the best frequency of injection site recordings were insufficient to resolve whether ascending projections of NA/NL, SO, and LLD were separated into microdomains or intermixed. In comparison, descending projections appeared to originate from all areas of TSC.

TSC projects to the ipsilateral superficial nucleus (TSS) and to the ipsilateral auditory thalamic nucleus medialis through the tractus opticus lateralis. The results reveal conserved patterns of brainstem auditory connections in lizards and archosaurs.

## 4 Discussion

The ascending auditory pathways of lizards resemble the well‐described mammalian and avian auditory pathways, from the level of the first‐order neurons in the VIIIth nerve to the telencephalon (Grothe et al. 2004; Wild et al. 2010). Foster and Hall (1978) first described these pathways in the green iguana, *Iguana iguana*, and we have observed similar and more differentiated connections in the Tokay gecko. Most geckos are nocturnal predators that use vocalizations for intraspecific communication (Marcellini 1977; Tang et al. 2001; Yu et al. 2011), with some species using loud (long distance) and others using quiet calls (short range) (Chen et al. 2016). It is therefore not surprising that geckos possess well‐developed auditory nuclei, including a large midbrain torus semicircularis with well‐delineated subdivisions and a complex superior olivary system.

### 4.1 Auditory Pathways in Lizard Brainstem

Among diapsids, the organization of ascending auditory circuits has been most frequently studied in birds (for reviews, see Ryugo and Parks [2003] and Grothe et al. [2004]). In this group, the auditory nerve projects to two cochlear nuclei, magnocellularis (NM) and angularis (NA) (Carr and Boudreau 1993; Parks and Rubel 1978). NM projects to the ipsilateral dorsal and contralateral ventral sides of NL, extracting the phase‐locked time information in NM and comparing it in NL (Carr and Konishi 1990). Both NL and NA project predominantly to the contralateral olivary nuclei, lemniscal nuclei, and the auditory midbrain, termed the nucleus of the inferior colliculus (Knudsen 1983; Krützfeldt et al. 2010b, Krützfeldt et al. 2010a; Takahashi and Konishi 1988a, 1988b) and also called the torus semicircularis and the nucleus mesencephalicus dorsalis (Covey and Carr 2005; Karten 1967; Puelles et al. 1994).

The major difference between the auditory systems of archosaurs and lizards may stem from the unique organization of the lizard inner ear (Manley 2023). During lizard evolution, the primitive papilla is thought to have consisted of three hair cell areas. The central (ancestral) hair cell patch responded to low frequencies, while the areas that flanked it contained hair cells that responded to higher frequency ranges (Manley 2023). The projections from the basilar papilla to the first‐order nuclei may reflect these evolutionary changes in the papilla. Auditory nerve projections have been described in a number of lizards, including geckos (Tang et al. 2012; Yan et al. 2010), alligator lizards (Szpir et al. 1990), iguanas (Foster and Hall 1978), savannah monitors (Barbas‐Henry and Lohman 1988), and tegus (DeFina and Webster 1974). An extra cochlear nucleus division, medial NA, has been described in these lizards, with a possible explanation provided by Szpir et al. (1990).

In alligator lizards, Szpir labeled and physiologically characterized individual auditory nerve axons (Szpir et al. 1990). These axons have different projection patterns depending on the best frequency. Lower‐best‐frequency fibers project to both NM and the lateral NA. High‐best‐frequency fibers project only to the medial NA. Szpir et al. (1990) concluded that different divisions of the cochlear nucleus are associated with separate frequency ranges and hypothesized that stimuli in the different frequency ranges were processed separately in the brain. A similar pattern was observed following small dye injections into the gecko auditory nerve (Tang et al. 2012). Lizards thus provide the only example where some auditory nerve fibers do not bifurcate to terminate in two first‐order nuclei, NA and NM (Ryugo and Parks 2003). Instead, in the two lizard species examined, high‐best‐frequency fibers only project to the medial NA, forming a unique high‐best‐frequency pathway proportionally larger than in other diapsids (Han et al. 2024).

The gecko NM projects bilaterally to both the ipsilateral and contralateral NL, as seen in other diapsids (Carr et al. 2009; Takahashi and Konishi 1988b; Tang et al. 2012; Willis and Carr 2017; Yan et al. 2010). Nevertheless, there remains uncertainty about whether NL was present in the shared ancestor of archosaurs and lepidosaurs, since NL has not been recognized in all lizards (Foster and Hall 1978; Miller 1975). The lizard NL may be difficult to find; it is small when compared to archosaur NL (Han et al. 2024; Tang et al. 2012; Yan et al. 2010), perhaps because the highly directional lizard periphery diminishes the need for central computation of sound source location (Christensen‐Dalsgaard et al. 2011, Christensen‐Dalsgaard et al. 2021; Christensen‐Dalsgaard and Carr 2018).

### 4.2 Nuclei of the Lateral Lemniscus in Tetrapods

The lemniscal nuclei in birds and geckos are both well developed, with similar numbers of nuclei. The birds studied so far (barn owl, zebra finch, and pigeon) have four nuclei of the lateral lemniscus: dorsal (LLD, divided into anterior and posterior divisions), intermediate (LLI), and ventral (LLV) (Arends and Zeigler 1986; Krützfeldt et al. 2010b; Leibler 1975; Takahashi and Konishi 1988a). In our previous study using antibodies against calcium‐binding and synaptic proteins, we had only recognized three lemniscal nuclei in the Tokay gecko, an LLD anterior and posterior (Yan et al. 2010), and a nucleus we homologized with the avian LLV, the ventral superior olive (SOv). We proposed that the avian LLV and gecko SOv were homologous, based on similar anatomical connections and positions in the brainstem (Yan et al. 2010). A fourth lemniscal nucleus in geckos, the medial LLD, was revealed in the present study with the use of neural tract tracing.

The avian and gecko lemniscal nuclei have similar connections. In geckos, LLDa and LLDp receive mainly ascending projections from NL, NA, and SOd, while LLDm largely receives ascending projections from SOd. In the zebra finch, Krützfeldt et al. (2010b) showed that LLD and LLV mainly receive ascending inputs from the cochlear nuclei, while LLI was a recipient of afferent fibers from SO. Thus, LLDm in the Tokay gecko and the avian LLI share similar ascending projections. In barn owl, zebra finch, and gecko, LLD was further characterized by reciprocal connections to the contralateral LLD via the commissure of Probst, and by ascending projections to the avian auditory midbrain (Takahashi and Keller 1992), like connections observed in the Tokay gecko. LLD also contains a preponderance of glutamate decarboxylase (GAD)‐positive cell bodies and large, often perisomatic, GAD‐positive terminals (Yan et al. 2010). This is of interest, given that the interconnected LLD mediates EI processing of interaural‐level differences in barn owls (Manley, Köppl, et al. 1988; Mogdans and Knudsen 1994; Takahashi and Keller 1992). In some birds, the posterior and anterior divisions of LLD receive distinct projections from the cochlear nuclei. The avian LLDp receives projects from NA, while LLDa receives inputs from NL in barn owl (Takahashi and Konishi 1988a) and zebra finch (Krützfeldt et al. 2010b), although these subdivisions could not be identified in chicks (Conlee and Parks 1986) and pigeons (Wild 1995). We could not determine whether a similar distinction exists between the projections from NA and NL in the Tokay gecko. Thus, although there are many similarities between the lemniscal complex in birds and geckos, projections at the single‐cell level remain unresolved.

Lemniscal nuclei are also present in frogs and mammals. In frogs, the lemniscal nuclei receive collaterals of ascending axons from the dorsal medullary nucleus and SO fibers and connect reciprocally to them (Feng 1986a, 1986b). They also project to the torus (Wilczynski 1981) and contain neurons responsive to acoustic stimuli (G. J. Rose and Wilczynski 1984). Unlike all other tetrapods studied to date, the lateral lemniscus in amphibians is a single nucleus lacking obvious subdivisions, although previous authors have speculated that cell clusters located within this nucleus may be analogous to the mammalian nuclei of the lateral lemniscus (Larsell 1934; Röthig 1927; Wilczynski and Endepols 2006). Mammals are generally regarded as possessing four lemniscal nuclei—ventral (two parts), intermediate, and dorsal nuclei (VNLL, INLL, and DNLL, respectively)—that differ in cytoarchitecture, connections, and immuno‐cytochemical properties (Schofield 2005). DNLL and VNLL are recognized across species. INLL is sometimes considered a dorsal division of VNLL, while VNLL is often further divided into two or more parts. Many VNLL and DNLL cells use glycine or GABA as a neurotransmitter, suggesting that they provide inhibitory projections to IC. Finally, the mammalian DNLL nuclei are reciprocally inhibited via the commissure of Probst (Schofield 2005), like the gecko LLD and the avian LLDp (Glendenning et al. 1981; Wild et al. 2010; Takahashi and Keller 1992). Thus, the nuclei of the lateral lemniscus in tetrapods share many features. In comparison with teleosts, which do not have lemniscal nuclei, we observe that tetrapod lemniscal nuclei exhibit similar subregions and connections in birds, geckos, and mammals.

### 4.3 Torus Semicircularis

The midbrain torus appears to be conserved through vertebrate evolution and to differentiate within each taxon (e.g., Aitkin 1986; Winer and Schreiner 2005). It acts as a junction to receive and send octavolateralis and mechanical information from and/or to the brainstem, diencephalon, and telencephalon. It is involved in acoustic information processing, specifically in directional hearing as well as analysis of complex sounds (Covey and Carr 2005; Edwards and Kelley 2001; Feng 1981). For amphibians, birds, and mammals, the torus appears to mostly mediate the extraction of acoustic features such as sound timing, intensity, frequency, and duration and to play an important role in both source localization and recognition (Bodnar and Bass 1997; Brand et al. 2000; Knudsen and Brainard 1991; Woolley and Casseday 2005). Examples of this diversity are reviewed below.

In frogs, the torus is an enlarged structure, containing five nuclei: laminar, principal, magnocellular, commissural, and subependymal (Potter 1965). Among them, the laminar, principal, and magnocellular subdivisions appear to mediate auditory processing, specifically directional hearing as well as analysis of complex sounds (Edwards and Kelley 2001; Elliott et al. 2011; Feng 1981; Feng et al. 1990; Hoke et al. 2004).

The avian torus has three divisions: the toral nucleus or the nucleus mesencephalicus lateralis pars dorsalis (MLd), the intercollicular nucleus, and the preisthmic superficial area. The terminology varies, with the toral nucleus also being called the central nucleus or the auditory midbrain (Aralla et al. 2024; Knudsen 1983; Puelles et al. 1994; Wagner et al. 2003; Wang and Karten 2010; Wild 1995). The toral/central nucleus is the major ascending auditory target of the brainstem auditory nuclei in birds and is tonotopically organized (Konishi and Knudsen 1978). The ascending inputs from the brainstem to the toral nucleus form distinct species‐typical projection zones in barn owls, chickens, and passerines (Aralla et al. 2024; Braun et al. 1985, Braun et al. 1991; Knudsen 1983; Krützfeldt et al. 2010a; Takahashi and Konishi 1988b; Wang and Karten 2010).

The mammalian inferior colliculus, homologous to the torus semicircularis in other species, has been divided into the central, dorsal, and external nuclei, which vary with lifestyle (Morest and Oliver 1984; Oliver and Shneiderman 1991; Rockel and Jones 1973a, 1973b). Inputs to the central nucleus form distinct species‐typical projection zones related to ecotype (Covey and Carr 2005; Winer and Schreiner 2005).

In lizards, the torus forms a complex of three nuclei: central, laminar, and superficial (Browner and Baruch 1984; Browner and Rubinson 1977; Kennedy and Browner 1981; ten Donkelaar et al. 1987). The central nucleus receives ascending auditory input, like the central nuclei in birds and mammals (Foster and Hall 1978; Kennedy 1974; Kennedy and Browner 1981). In different lizard species, the boundaries and definition of the laminar nucleus (dorsal nucleus in Foster and Hall 1978) vary among authors, and their associated connections and implied function also differ (e.g., Butler and Bruce 1981; ten Donkelaar and de Boer‐van Huizen 1987; see Díaz et al. [2000] for discussion). In the Tokay gecko, the laminar nucleus is associated with vocalization (Kennedy 1975) and has been compared to the avian intercollicular nucleus (Puelles et al. 1994). The superficial nucleus, also referred to as the lacertian intercollicular nucleus (Ebbesson 1967; Foster and Hall 1978; ten Donkelaar et al. 1987) and not to be confused with the avian intercollicular nucleus, is formed by the central stratum and mainly receives ascending spinal input, comparable to the avian preisthmic superficial area (Díaz et al. 2000; Puelles et al. 1994). Foster and Hall (1978) noted degeneration in the superficial nucleus following lesions in TSC, and similar connections were observed in our study. The superficial nucleus is likely homologous to the paratorus in snakes based on location and connections (Senn 1969; Senn and Northcutt 1973; Han personal observations).

The lizard central nucleus, like that of birds and mammals, may be subdivided into several regions based upon both cytoarchitecture and connections. We had used the spatial pattern of calcium‐binding protein expression to divide the gecko TSC into three subregions—lateral, ventral, and dorsal (Yan et al. 2010). In the present study, we revised our previous conclusion and separated TSC into only two subdivisions—TSCv and TSCd—for two reasons: (1) “Lateral” TSC was defined by the abundance of calretinin‐positive fibers, likely from ascending calretinin‐positive neurons in the acoustic tubercle, which also extend into TSCv and to a lesser extent TSCd, and (2) the separation of TSC into TSCv and TSCd is supported by Nissl staining, Golgi staining, as well as parvalbumin immunostaining. Furthermore, the distribution of ascending projections supports the separation into dorsal and ventral subdivisions. Projections from the acoustic tubercle (NA, NL, and VeO) match the distribution of calretinin‐positive fibers and parvalbumin‐positive neuropil in TSC (Yan et al. 2010), with a dorsal–ventral organization of saccular (VeO) and cochlear input (NA/NL).

Evaluation of dye injections into TSC did not reveal a straightforward tonotopic organization. Instead, different regions in TSCv were associated with high‐ or low‐best‐frequency responses and may reflect parallel ascending high‐ or low‐best‐frequency channels from the olivary complex and acoustic tubercle. Absence of evidence is not evidence of absence, however, and regular changes in best frequency, or tonotopy, were observed at one caudal and medial recording site in TSCv, suggesting that tonotopic organization might be present in a restricted location in gecko TSCv. Reconstruction of the other injection sites in TSC did not reveal the tonotopic organization that characterizes the auditory midbrain of birds and crocodilians (Konishi and Knudsen 1978; Manley 1971) and the mammalian inferior colliculus (Grinnell 1963; J. E. Rose et al. 1963). Additional studies will be needed to resolve details of the response types, projections, and organization of TSC, including the directional processing mentioned below.

In TSCd, we typically observed responses to more than one frequency. The multiunit recording from dye injection electrodes precluded unambiguous separation of responses into bimodal units or a mixture of units tuned to either high or low best frequencies. It is likely that some units were bimodal, since complex tuning curves were also recorded in the only previous study of physiological responses from the Tokay torus (Sammaritano‐Klein 1976, also in Manley 1981). Sammaritano‐Klein and Manley recorded from 87 neurons in TS in the Tokay, with CFs ranging from 100 to 4700 Hz, and observed both V‐shaped and more complex tuning curves. In our study, units recorded in TSCd responses were typically broad or bimodal, reflecting the distribution of responses in the gecko auditory nerve, with regions of greatest sensitivity in both low (around 300 Hz) and high best frequencies (around 2000 Hz) (Christensen‐Dalsgaard et al. 2021). TSCv receives inputs from TSCd, which in turn projects to both the brainstem and the nucleus medialis of the thalamus, suggesting that further processing may take place within TS. Sammaritano‐Klein and Manley (1976) reported directional responses, mostly to sound from contralateral directions. These free field responses are comparable to those recorded from auditory nerve fibers (Christensen‐Dalsgaard et al. 2021).

In summary, the location, basic structures, and connections of the torus appear largely unchanged during vertebrate evolution, while its anatomical complexity may reflect the varied demands of the auditory system. The well‐developed gecko torus might mediate both sound localization and call recognition, like in other land vertebrates.

## Author Contributions

All authors participated in conceptualization and design of the study. Dawei Han, Yezhong Tang, Wenru Liang, Jakob Christensen‐Dalsgaard and Catherine E. Carr conducted experiments, performed data analysis, and prepared figures. Dawei Han, Yezhong Tang, and Catherine E. Carr wrote the original draft. Dawei Han, Jakob Christensen‐Dalsgaard, and Catherine E. Carr reviewed and edited the manuscript. Yezhong Tang, Jakob Christensen‐Dalsgaard, and Catherine E. Carr provided resources and funding.

## Peer Review

The peer review history for this article is available at https://publons.com/publon/10.1002/cne.70100

## Data Availability

The data that support the findings of this study are available from the corresponding author upon reasonable request.

## References

[cne70100-bib-0001] Aitkin, L. 1986. The Auditory Midbrain: Structure and Function in the Central Auditory Pathway. Humana Press. 10.1007/978-1-59259-460-3.

[cne70100-bib-0002] Aralla, R. , C. Pauley , and C. Köppl . 2024. “The Neural Representation of Binaural Sound Localization Cues Across Different Subregions of the Chicken's Inferior Colliculus.” Journal of Comparative Neurology 532, no. 7: e25653. 10.1002/cne.25653. 38962885

[cne70100-bib-0003] Arends, J. J. A. , and H. P. Zeigler . 1986. “Anatomical Identification of an Auditory Pathway From a Nucleus of the Lateral Lemniscal System to the Frontal Telencephalon (Nucleus Basalis) of the Pigeon.” Brain Research 398, no. 2: 375–381. 10.1016/0006-8993(86)91499-X. 3801910

[cne70100-bib-0004] Barbas‐Henry, H. A. , and A. H. M. Lohman . 1988. “Primary Projections and Efferent Cells of the VIIIth Cranial Nerve in the Monitor Lizard, Varanus Exanthematicus.” Journal of Comparative Neurology 277, no. 2: 234–249. 10.1002/cne.902770206. 2466058

[cne70100-bib-0005] Bodnar, D. A. , and A. H. Bass . 1997. “Temporal Coding of Concurrent Acoustic Signals in Auditory Midbrain.” Journal of Neuroscience 17, no. 19: 7553–7564. 10.1523/jneurosci.17-19-07553.1997. 9295400 PMC6573432

[cne70100-bib-0006] Brand, A. , A. Urban , and B. Grothe . 2000. “Duration Tuning in the Mouse Auditory Midbrain.” Journal of Neurophysiology 84, no. 4: 1790–1799. 10.1152/jn.2000.84.4.1790. 11024071

[cne70100-bib-0007] Braun, K. , H. Scheich , C. W. Heizmann , and W. Hunziker . 1991. “Parvalbumin and Calbindin‐D28K Immunoreactivity as Developmental Markers of Auditory and Vocal Motor Nuclei of the Zebra Finch.” Neuroscience 40, no. 3: 853–869. 10.1016/0306-4522(91)90017-I. 2062443

[cne70100-bib-0008] Braun, K. , H. Scheich , M. Schachner , and C. W. Heizmann . 1985. “Distribution of Parvalbumin, Cytochrome Oxidase Activity and 14C‐2‐Deoxyglucose Uptake in the Brain of the Zebra Finch—II. Visual System.” Cell and Tissue Research 240, no. 1: 117–127. 10.1007/BF00217564.

[cne70100-bib-0009] Browner, R. H. , and A. M. Baruch . 1984. “The Cytoarchitecture of the Torus Semicircularis in the Golden Skink *Mabuya multifasciata* .” Journal of Morphology 180, no. 3: 223–242. 10.1002/jmor.1051800306. 30037159

[cne70100-bib-0010] Browner, R. H. , and K. Rubinson . 1977. “The Cytoarchitecture of the Torus Semicircularis in the Tegu Lizard, *Tupinambis nigropunctatus* .” Journal of Comparative Neurology 176, no. 4: 539–557. 10.1002/cne.901760406. 303647

[cne70100-bib-0011] Butler, A. B. , and L. L. Bruce . 1981. “Nucleus Laminaris of the Torus Semicircularis: Projection to Spinal Cord in Reptiles.” Neuroscience Letters 25, no. 3: 221–225. 10.1016/0304-3940(81)90395-5. 6270598

[cne70100-bib-0012] Carr, C. E. , and R. E. Boudreau . 1993. “Organization of the Nucleus Magnocellularis and the Nucleus Laminaris in the Barn Owl: Encoding and Measuring Interaural Time Differences.” Journal of Comparative Neurology 334, no. 3: 337–355. 10.1002/cne.903340302. 8376623

[cne70100-bib-0013] Carr, C. E. , and J. Christensen‐Dalsgaard . 2016. “Evolutionary Trends in Directional Hearing.” Current Opinion in Neurobiology 40: 111–117. 10.1016/j.conb.2016.07.001. 27448850 PMC5056815

[cne70100-bib-0014] Carr, C. E. , and R. A. Code . 2000. “The central Auditory System of Reptiles and Birds.” In Comparative Hearing: Birds and Reptiles, edited by R. J. Dooling , R. R. Fay , and A. N. Popper , 197–248. Springer. 10.1007/978-1-4612-1182-2_5.

[cne70100-bib-0015] Carr, C. E. , and P. L. Edds‐Walton . 2008. “Vertebrate Auditory Pathways.” In The Senses: A Comprehensive Reference. Vol. 3, edited by P. Dallos and D. Oertel , 499–523. Elsevier. 10.1016/B978-012370880-9.00041-4.

[cne70100-bib-0016] Carr, C. E. , and M. Konishi . 1990. “A Circuit for Detection of Interaural Time Differences in the Brain Stem of the Barn Owl.” Journal of Neuroscience 10, no. 10: 3227–3246. 10.1523/jneurosci.10-10-03227.1990. 2213141 PMC6570189

[cne70100-bib-0017] Carr, C. E. , D. Soares , J. Smolders , and J. Z. Simon . 2009. “Detection of Interaural Time Differences in the Alligator.” Journal of Neuroscience 29, no. 25: 7978–7982. 10.1523/JNEUROSCI.6154-08.2009. 19553438 PMC3170862

[cne70100-bib-0018] Chen, J. , T. Jono , J. Cui , X. Yue , and Y. Tang . 2016. “The Acoustic Properties of Low Intensity Vocalizations Match Hearing Sensitivity in the Webbed‐Toed Gecko, *Gekko subpalmatus* .” PLoS ONE 11, no. 1: e0146677. 10.1371/journal.pone.0146677. 26752301 PMC4709187

[cne70100-bib-0019] Christensen‐Dalsgaard, J. , and C. Carr . 2018. “Processing of Directional Information in the Gecko Auditory Nerve.” Acta Acustica United with Acustica 104, no. 5: 848–851. 10.3813/AAA.919242.

[cne70100-bib-0020] Christensen‐Dalsgaard, J. , P. Kuokkanen , J. E. Matthews , and C. E. Carr . 2021. “Strongly Directional Responses to Tones and Conspecific Calls in the Auditory Nerve of the Tokay Gecko, *Gekko gecko* .” Journal of Neurophysiology 125, no. 3: 887–902. 10.1152/jn.00576.2020. 33534648 PMC7988750

[cne70100-bib-0021] Christensen‐Dalsgaard, J. , Y. Tang , and C. E. Carr . 2011. “Binaural Processing by the Gecko Auditory Periphery.” Journal of Neurophysiology 105, no. 5: 1992–2004. 10.1152/jn.00004.2011. 21325679 PMC3094191

[cne70100-bib-0022] Clack, J. A. 1997. “The Evolution of Tetrapod Ears and the Fossil Record.” Brain, Behavior and Evolution 50, no. 4: 198–212. 10.1159/000113334. 9310195

[cne70100-bib-0023] Clack, J. A. 2015. “Evolutionary Biology: The Origin of Terrestrial Hearing.” Nature 519, no. 7542: 168–169. 25762279 10.1038/519168a

[cne70100-bib-0024] Conlee, J. W. , and T. N. Parks . 1986. “Origin of Ascending Auditory Projections to the Nucleus Mesencephalicus Lateralis Pars Dorsalis in the Chicken.” Brain Research 367, no. 1–2: 96–113. 10.1016/0006-8993(86)91583-0. 3697720

[cne70100-bib-0025] Covey, E. , and C. E. Carr . 2005. “The Auditory Midbrain in Bats and Birds.” In The Inferior Colliculus, edited by J. A. Winer and C. E. Schneider , 493–536. Wiley. 10.1007/0-387-27083-3_17.

[cne70100-bib-0026] DeFina, A. V. , and D. B. Webster . 1974. “Projections of the Intraotic Ganglion to the Medullary Nuclei in the Tegu Lizard, *Tupinambis nigropunctatus* .” Brain, Behavior and Evolution 10, no. 1–3: 197–211. 10.1159/000124312. 4141917

[cne70100-bib-0027] Díaz, C. , C. Yanes , C. M. Trujillo , and L. Puelles . 2000. “Cytoarchitectonic Subdivisions in the Subtectal Midbrain of the Lizard *Gallotia galloti* .” Journal of Neurocytology 29, no. 8: 569–593. 10.1023/A:1011067918585. 11283413

[cne70100-bib-0028] Ebbesson, S. O. E. 1967. “Ascending Axon Degeneration Following Hemisection of the Spinal Cord in the tegu Lizard (*Tupinambis nigropunctatus*).” Brain Research 5, no. 2: 178–206. 10.1016/0006-8993(67)90086-8. 6033146

[cne70100-bib-0029] Edwards, C. J. , and D. B. Kelley . 2001. “Auditory and Lateral Line Inputs to the Midbrain of an Aquatic Anuran; Neuroanatomic Studies in *Xenopus laevis* .” Journal of Comparative Neurology 438, no. 2: 148–162. 10.1002/cne.1306. 11536185 PMC3493254

[cne70100-bib-0030] Elliott, T. M. , J. Christensen‐Dalsgaard , and D. B. Kelley . 2011. “Temporally Selective Processing of Communication Signals by Auditory Midbrain Neurons.” Journal of Neurophysiology 105, no. 4: 1620–1632. 10.1152/jn.00261.2009. 21289132 PMC3075307

[cne70100-bib-0031] Feng, A. S. 1981. “Directional Response Characteristics of Single Neurons in the Torus Semicircularis of the Leopard Frog (*Rana pipiens*).” Journal of Comparative Physiology A 144, no. 3: 419–428. 10.1007/BF00612574.

[cne70100-bib-0032] Feng, A. S. 1986a. “Afferent and Efferent Innervation Patterns of the Cochlear Nucleus (Dorsal Medullary Nucleus) of the Leopard Frog.” Brain Research 367, no. 1–2: 183–191. 10.1016/0006-8993(86)91591-X. 3486022

[cne70100-bib-0033] Feng, A. S. 1986b. “Afferent and Efferent Innervation Patterns of the Superior Olivary Nucleus of the Leopard Frog.” Brain Research 364, no. 1: 167–171. 10.1016/0006-8993(86)90998-4. 3484990

[cne70100-bib-0034] Feng, A. S. , J. C. Hall , and D. M. Gooler . 1990. “Neural Basis of Sound Pattern Recognition in Anurans.” Progress in Neurobiology 34, no. 4: 313–329. 10.1016/0301-0082(90)90008-5. 2185497

[cne70100-bib-0035] Feng, A. S. , and W. Lin . 1991. “Differential Innervation Patterns of Three Divisions of Frog Auditory Midbrain (Torus Semicircularis).” Journal of Comparative Neurology 306, no. 4: 613–630. 10.1002/cne.903060407. 1712796

[cne70100-bib-0036] Foster, R. E. , and W. C. Hall . 1978. “The Organization of central Auditory Pathways in a Reptile, *Iguana iguana* .” Journal of Comparative Neurology 178, no. 4: 783–831. 10.1002/cne.901780412. 632382

[cne70100-bib-0037] Frankenberg, E. 1982. “Vocal Behavior of the Mediterranean House Gecko, *Hemidactylus turcicus* .” Copeia 1982, no. 4: 770–775. 10.2307/1444085.

[cne70100-bib-0038] Fritzsch, B. 1992. “The Water‐to‐Land Transition: Evolution of the Tetrapod Basilar Papilla, Middle Ear, and Auditory Nuclei.” In The Evolutionary Biology of Hearing, edited by D. B. Webster , A. N. Popper , and R. R. Fay , 351–375. Springer. 10.1007/978-1-4612-2784-7_22.

[cne70100-bib-0039] Glaser, E. M. , and H. Van der Loos . 1981. “Analysis of Thick Brain Sections by Obverse‐Reverse Computer Microscopy: Application of a New, High Clarity Golgi‐Nissl Stain.” Journal of Neuroscience Methods 4, no. 2: 117–125. 10.1016/0165-0270(81)90045-5. 6168870

[cne70100-bib-0040] Glendenning, K. K. , J. K. Brusno‐Bechtold , G. C. Thompson , and R. B. Masterton . 1981. “Ascending Auditory Afferents to the Nuclei of the Lateral Leminscus.” Journal of Comparative Neurology 197, no. 4: 673–703. 10.1002/cne.901970409. 7229133

[cne70100-bib-0041] Grinnell, A. D. 1963. “The Neurophysiology of Audition in Bats: Intensity and Frequency Parameters.” The Journal of Physiology 167, no. 1: 38–66. 10.1113/jphysiol.1963.sp007132. 13950553 PMC1359484

[cne70100-bib-0042] Grothe, B. , C. E. Carr , J. H. Casseday , B. Fritzsch , and C. Köppl . 2004. “The Evolution of Central Pathways and Their Neural Processing Patterns.” In Evolution of the Vertebrate Auditory System, edited by G. A. Manley , A. N. Popper , and R. R. Fay , 289–359. Springer. 10.1007/978-1-4419-8957-4_10.

[cne70100-bib-0043] Han, D. , and C. E. Carr . 2024. “Auditory Pathway for Detection of Vibration in the Tokay Gecko.” Current Biology 34, no. 21: 4908.e3–4919.e3. 10.1016/j.cub.2024.09.016. 39368471 PMC11537832

[cne70100-bib-0044] Han, D. , R. W. Fuquen , K. L. Willis , J. Christensen‐Dalsgaard , and C. E. Carr . 2024. “Sound Localization Circuits in Reptiles.” Frontiers in Amphibian and Reptile Science 2: 1429172. 10.3389/famrs.2024.1429172.

[cne70100-bib-0045] Hibbitts, T. J. , M. J. Whiting , and D. M. Stuart‐Fox . 2007. “Shouting the Odds: Vocalization Signals Status in a Lizard.” Behavioral Ecology and Sociobiology 61, no. 8: 1169–1176. 10.1007/s00265-006-0330-x.

[cne70100-bib-0046] Hoke, K. L. , S. S. Burmeister , R. D. Fernald , A. S. Rand , M. J. Ryan , and W. Wilczynski . 2004. “Functional Mapping of the Auditory Midbrain During Mate Call Reception.” Journal of Neuroscience 24: 11264–11272. 15601932 10.1523/JNEUROSCI.2079-04.2004 PMC6730357

[cne70100-bib-0047] Horowitz, S. S. , J. A. Chapman , and A. M. Simmons . 2006. “Plasticity of Auditory Medullary‐Midbrain Connectivity Across Metamorphic Development in the Bullfrog, *Rana catesbeiana* .” Brain, Behavior and Evolution 69, no. 1: 1–19. 10.1159/000095027. 16912473 PMC3257804

[cne70100-bib-0048] Ito, T. , and M. S. Malmierca . 2018. “Neurons, Connections, and Microcircuits of the Inferior Colliculus.” In The Mammalian Auditory Pathways: Synaptic Organization and Microcircuits, edited by D. L. Oliver , N. B. Cant , R. R. Fay , and A. N. Popper , 127–167. Springer. 10.1007/978-3-319-71798-2_6.

[cne70100-bib-0049] Jono, T. , and Y. Inui . 2012. “Secret Calls From Under the Eaves: Acoustic Behavior of the Japanese House Gecko, *Gekko japonicus* .” Copeia 1: 145–149. 10.1643/CE-10-169.

[cne70100-bib-0050] Karten, H. J. 1967. “The Organization of the Ascending Auditory Pathway in the Pigeon (*Columba livia*) I. Diencephalic Projections of the Inferior Colliculus (Nucleus Mesencephali Lateralis, Pars Dorsalis).” Brain Research 6, no. 3: 409–427. 10.1016/0006-8993(67)90055-8. 6076249

[cne70100-bib-0051] Kennedy, M. C. 1974. “Auditory Multiple‐Unit Activity in the Midbrain of the Tokay Gecko (*Gekko gecko*, L.).” Brain, Behavior and Evolution 10, no. 1–3: 257–264. 10.1159/000124317. 4455355

[cne70100-bib-0052] Kennedy, M. C. 1975. “Vocalization Elicited in a Lizard by Electrical Stimulation of the Midbrain.” Brain Research 91, no. 2: 321–325. 10.1016/0006-8993(75)90556-9. 1164681

[cne70100-bib-0053] Kennedy, M. C. , and R. H. Browner . 1981. “The Torus Semicircularis in a Gekkonid Lizard.” Journal of Morphology 169, no. 3: 259–274. 10.1002/jmor.1051690302. 30153716

[cne70100-bib-0054] Knudsen, E. I. 1983. “Subdivisions of the Inferior Colliculus in the Barn Owl (*Tyto alba*).” Journal of Comparative Neurology 218, no. 2: 174–186. 10.1002/cne.902180205. 6886070

[cne70100-bib-0055] Knudsen, E. I. , and M. S. Brainard . 1991. “Visual Instruction of the Neural Map of Auditory Space in the Developing Optic Tectum.” Science 253, no. 5015: 85–87. 10.1126/science.2063209. 2063209

[cne70100-bib-0056] Konishi, M. , and E. I. Knudsen . 1978. “A Neural Map of Auditory Space in the Owl.” Science 200, no. 4343: 795–797. 10.1126/science.644324. 644324

[cne70100-bib-0057] Krützfeldt, N. O. E. , P. Logerot , M. F. Kubke , and J. M. Wild . 2010a. “Connections of the Auditory Brainstem in a Songbird, *Taeniopygia guttata*. I. Projections of Nucleus Angularis and Nucleus Laminaris to the Auditory Torus.” Journal of Comparative Neurology 518, no. 11: 2109–2134. 10.1002/cne.22334. 20394061 PMC3862038

[cne70100-bib-0058] Krützfeldt, N. O. E. , P. Logerot , M. F. Kubke , and J. M. Wild . 2010b. “Connections of the Auditory Brainstem in a Songbird, *Taeniopygia guttata*. II. Projections of Nucleus Angularis and Nucleus Laminaris to the Superior Olive and Lateral Lemniscal Nuclei.” Journal of Comparative Neurology 518, no. 11: 2135–2148. 10.1002/cne.22324. 20394062 PMC3862037

[cne70100-bib-0059] Larsell, O. 1934. “The Differentiation of the Peripheral and Central Acoustic Apparatus in the Frog.” Journal of Comparative Neurology 60, no. 3: 473–527. 10.1002/cne.900600306.

[cne70100-bib-0060] Leibler, L. M. 1975. “Monaural and Binaural Pathways in the Ascending Auditory System of the Pigeon.” Doctoral diss., Massachusetts Institute of Technology. http://scholar.google.com/scholar?hl=en&btnG=Search&q=intitle:Monaural+and+binaural+pathways+in+the+ascending+auditory+system+of+the+pigeon#0.

[cne70100-bib-0061] Manley, G. A. 1981. “A Review of the Auditory Physiology of the Reptiles.” Progress in Sensory Physiology 2: 49–134. 10.1007/978-3-642-68169-1_2.

[cne70100-bib-0062] Manley, G. A. 2023. “Evolution of Diversity in the Auditory Papillae of Reptiles.” Diversity 15, no. 6: 730. 10.3390/d15060730.

[cne70100-bib-0063] Manley, G. A. , and O. Gleich . 1992. “Evolution and Specialization of Function in the Avian Auditory Periphery.” In The Evolutionary Biology of Hearing, edited by D. B. Webster , R. R. Fay , and A. N. Popper , 561–580. Springer‐Verlag. 10.1007/978-1-4612-2784-7_34.

[cne70100-bib-0064] Manley, G. A. , C. Köppl , and M. Konishi . 1988. “A Neural Map of Interaural Intensity Differences in the Brain Stem of the Barn Owl.” Journal of Neuroscience 8, no. 8: 2665–2676. 10.1523/jneurosci.08-08-02665.1988. 3411346 PMC6569385

[cne70100-bib-0065] Manley, G. A. , G. K. Yates , and C. Köppl . 1988. “Auditory Peripheral Tuning: Evidence for a Simple Resonance Phenomenon in the Lizard Tiliqua.” Hearing Research 33, no. 2: 181–189. 10.1016/0378-5955(88)90031-7. 3397328

[cne70100-bib-0066] Manley, J. A. 1971. “Single Unit Studies in the Midbrain Auditory Area of Caiman.” Zeitschrift Für Vergleichende Physiologie 71, no. 3: 255–261. 10.1007/BF00298138.

[cne70100-bib-0067] Marcellini, D. 1977. “Acoustic and Visual Display Behavior of Gekkonid Lizards.” American Zoologist 17, no. 1: 251–260. 10.1093/icb/17.1.251.

[cne70100-bib-0068] Miller, M. R. 1975. “The Cochlear Nuclei of Lizards.” Journal of Comparative Neurology 159, no. 3: 375–406. 10.1002/cne.901590306. 1112916

[cne70100-bib-0069] Mogdans, J. , and E. I. Knudsen . 1994. “Representation of Interaural Level Difference in the VLVp, the First Site of Binaural Comparison in the Barn Owl's Auditory System.” Hearing Research 74, no. 1–2: 148–164. 10.1016/0378-5955(94)90183-X. 8040085

[cne70100-bib-0070] Morest, D. K. , and D. L. Oliver . 1984. “The Neuronal Architecture of the Inferior Colliculus in the Cat: Defining the Functional Anatomy of the Auditory Midbrain.” Journal of Comparative Neurology 222, no. 2: 209–236. 10.1002/cne.902220206. 6699208

[cne70100-bib-0071] Northcutt, R. G. 1980. “Central Auditory Pathways in Anamniotic Vertebrates.” In Comparative Studies of Hearing in Vertebrates, edited by A. N. Popper and R. R. Fay , 79–118. Springer. 10.1007/978-1-4613-8074-0_3.

[cne70100-bib-0072] Oliver, D. L. 2005. “Neuronal Organization in the Inferior Colliculus.” In The Inferior Colliculus, edited by J. A. Winer and C. E. Schreiner , 69–114. Springer. 10.1007/0-387-27083-3_2.

[cne70100-bib-0073] Oliver, D. L. , and A. Shneiderman . 1991. “The Anatomy of the Inferior Colliculus. A Cellular Basis for Integration of Monaural and Binaural Information.” Neurobiology of Hearing 2: 195–222.

[cne70100-bib-0074] Parks, T. N. , and E. W. Rubel . 1978. “Organization and Development of the Brain Stem Auditory Nuclei of the Chicken: Primary Afferent Projections.” Journal of Comparative Neurology 180, no. 3: 439–448. 10.1002/cne.901800303. 659669

[cne70100-bib-0075] Potter, H. D. 1965. “Mesencephalic Auditory Region of the Bullfrog.” Journal of Neurophysiology 28, no. 6: 1132–1154. 10.1152/jn.1965.28.6.1132. 5327587

[cne70100-bib-0076] Puelles, L. , C. Robles , M. Martínez‐de‐la‐Torre , and S. Martínez . 1994. “New Subdivision Schema for the Avian Torus Semicircularis: Neurochemical Maps in the Chick.” Journal of Comparative Neurology 340, no. 1: 98–125. 10.1002/cne.903400108. 8176005

[cne70100-bib-0077] Ramón‐Moliner, E. 1970. “The Golgi‐Cox Technique.” In Contemporary Methods in Neuroanatomy, edited by W. Nauta and S. Ebbesson , 32–55. Springer.

[cne70100-bib-0078] Rockel, A. J. , and E. G. Jones . 1973a. “The Neuronal Organization of the Inferior Colliculus of the Adult Cat. I. The Central Nucleus.” Journal of Comparative Neurology 147, no. 1: 11–59. 10.1002/cne.901470103. 4682181

[cne70100-bib-0079] Rockel, A. J. , and E. G. Jones . 1973b. “The Neuronal Organization of the Inferior Colliculus of the Adult Cat. II. The Pericentral Nucleus.” Journal of Comparative Neurology 149, no. 3: 301–333. 10.1002/cne.901490303. 4123504

[cne70100-bib-0080] Rose, G. J. , and W. Wilczynski . 1984. “The Anuran Superficial Reticular Nucleus: Evidence for Homology With Nuclei of the Lateral Lemniscus.” Brain Research 304, no. 1: 170–172. 10.1016/0006-8993(84)90876-X. 6611192

[cne70100-bib-0081] Rose, J. E. , D. D. Greenwood , J. M. Goldberg , and J. E. Hind . 1963. “Some Discharge Characteristics of Single Neurons in the Inferior Colliculus of the Cat. I. Tonotopical Organization, Relation of Spike‐Counts to Tone Intensity, and Firing Patterns of Single Elements.” Journal of Neurophysiology 26, no. 2: 294–320. 10.1152/jn.1963.26.2.294. 13954634

[cne70100-bib-0082] Röthig, P. 1927. “Beiträge Zum Studium des Zentralnervensystems Der Wirbeltiere. XI. Über die Faserzüge im Mittelhirn, Kleinhirn und der Medulla Oblongata Der Urodelen und Anuren.” Zeitschrift Fur Mikroskopisch‐Anatomische Forschung 10: 381–472.

[cne70100-bib-0083] Ryugo, D. K. , and T. N. Parks . 2003. “Primary Innervation of the Avian and Mammalian Cochlear Nucleus.” Brain Research Bulletin 60, no. 5–6: 435–456. 10.1016/S0361-9230(03)00049-2. 12787866

[cne70100-bib-0084] Sammaritano‐Klein, M. R. 1976. “Single Unit Responses in the Midbrain Auditory Nucleus of the Lizard Gekko Gecko.” Master's thesis, McGill University.

[cne70100-bib-0085] Sammaritano‐Klein, M. R. , and G. A. Manley . 1976. “Auditory Responses of Single Neurons in the Midbrain of the Tokay Gecko.” The Journal of the Acoustical Society of America 59, no. S1: S46–S47. 10.1121/1.2002719.

[cne70100-bib-0086] Schofield, B. R. 2005. “Superior Olivary Complex and Lateral Lemniscal Connections of the Auditory Midbrain.” In The Inferior Colliculus, edited by J. A. Winer and C. E. Schreiner , 132–154. Springer. 10.1007/0-387-27083-3_4.

[cne70100-bib-0087] Schwartz, I. R. 1992. “The Superior Olivary Complex and Lateral Lemniscal Nuclei.” In The Mammalian Auditory Pathway: Neuroanatomy, edited by D. B. Webster , A. N. Popper , and R. R. Fay , 117–167. Springer. 10.1007/978-1-4612-4416-5_4.

[cne70100-bib-0088] Senn, D. G. 1969. “The Saurian and Ophidian Colliculi Posteriores of the Midbrain.” Cells Tissues Organs 74, no. 1: 114–120. 10.1159/000143368. 5374931

[cne70100-bib-0089] Senn, D. G. , and R. G. Northcutt . 1973. “The Forebrain and Midbrain of Some Squamates and Their Bearing on the Origin of Snakes.” Journal of Morphology 140, no. 2: 135–151. 10.1002/jmor.1051400202. 4711260

[cne70100-bib-0090] Szpir, M. R. , S. Sento , and D. K. Ryugo . 1990. “Central Projections of Cochlear Nerve Fibers in the Alligator Lizard.” Journal of Comparative Neurology 295, no. 4: 530–547. 10.1002/cne.902950403. 2358519

[cne70100-bib-0091] Takahashi, T. T. , and C. H. Keller . 1992. “Commissural Connections Mediate Inhibition for the Computation of Interaural Level Difference in the Barn Owl.” Journal of Comparative Physiology A 170, no. 2: 161–169. 10.1007/BF00196898. 1374800

[cne70100-bib-0092] Takahashi, T. T. , and M. Konishi . 1988a. “Projections of Nucleus Angularis and Nucleus Laminaris to the Lateral Lemniscal Nuclear Complex of the Barn Owl.” Journal of Comparative Neurology 274, no. 2: 212–238. 10.1002/cne.902740207. 2463287

[cne70100-bib-0093] Takahashi, T. T. , and M. Konishi . 1988b. “Projections of the Cochlear Nuclei and Nucleus Laminaris to the Inferior Colliculus of the Barn Owl.” Journal of Comparative Neurology 274, no. 2: 190–211. 10.1002/cne.902740206. 2463286

[cne70100-bib-0094] Takahashi, T. T. , A. Moiseff , and M. Konishi . 1984. “Time and Intensity Cures Are Processed Independently in the Auditory System of the Owl.” Journal of Neuroscience 4, no. 7: 1781–1786. 10.1523/jneurosci.04-07-01781.1984. 6737040 PMC6564890

[cne70100-bib-0095] Tang, Y. , J. Christensen‐Dalsgaard , and C. E. Carr . 2012. “Organization of the Auditory Brainstem in a Lizard, *Gekko gecko*. I. Auditory Nerve, Cochlear Nuclei, and Superior Olivary Nuclei.” Journal of Comparative Neurology 520, no. 8: 1784–1799. 10.1002/cne.23013. 22120438 PMC4300985

[cne70100-bib-0096] Tang, Y. , L. Z. Zhuang , and Z. W. Wang . 2001. “Advertisement Calls and Their Relation to Reproductive Cycles in *Gekko gecko* (Reptilia, Lacertilia).” Copeia 1: 248–253. 10.1643/0045-8511(2001)001[0248:ACATRT]2.0.CO;2.

[cne70100-bib-0097] ten Donkelaar, H. J. , G. C. Bangma , H. A. Barbas‐Henry , R. de Boer‐van Huizen , and J. G. Wolters . 1987. “The Brain Stem in a Lizard, *Varanus exanthematicus* .” Advances in Anatomy, Embryology, and Cell Biology 107: 1–168. 3318284 10.1007/978-3-642-72763-4

[cne70100-bib-0098] ten Donkelaar, H. J. , and R. de Boer‐van Huizen . 1987. “A Possible Pain Control System in a Non‐Mammalian Vertebrate (a Lizard, *Gekko gecko*).” Neuroscience Letters 83, no. 1–2: 65–70. 10.1016/0304-3940(87)90217-5. 2831478

[cne70100-bib-0099] Wagner, H. , O. Güntürkün , and B. Nieder . 2003. “Anatomical Markers for the Subdivisions of the Barn Owl's Inferior‐Collicular Complex and Adjacent Peri‐ and Subventricular Structures.” Journal of Comparative Neurology 465, no. 1: 145–159. 10.1002/cne.10826. 12926022

[cne70100-bib-0100] Walkowiak, W. 1988. “Central Temporal Encoding.” In Evolution of the Amphibian Auditory System, edited by B. Fritzsch , M. J. Ryan , W. Wilczynski , T. E. Hetherington , and W. Walkowiak , 275–509. Wiley.

[cne70100-bib-0101] Walton, P. L. , J. Christensen‐Dalsgaard , and C. E. Carr . 2017. “Evolution of Sound Source Localization Circuits in the Nonmammalian Vertebrate Brainstem.” Brain, Behavior and Evolution 90, no. 2: 131–153. 10.1159/000476028. 28988244 PMC5691234

[cne70100-bib-0102] Wang, Y. , and H. J. Karten . 2010. “Three Subdivisions of the Auditory Midbrain in Chicks (*Gallus gallus*) Identified by Their Afferent and Commissural Projections.” Journal of Comparative Neurology 518, no. 8: 1199–1219. 10.1002/cne.22269. 20148439 PMC2878180

[cne70100-bib-0103] Wilczynski, W. 1981. “Afferents to the Midbrain Auditory Center in the Bullfrog, *Rana catesbeiana* .” Journal of Comparative Neurology 198, no. 3: 421–433. 10.1002/cne.901980304. 6972387

[cne70100-bib-0104] Wilczynski, W. , and H. Endepols . 2006. “Central Auditory Pathways in Anuran Amphibians: The Anatomical Basis of Hearing and Sound Communication.” In Hearing and Sound Communication in Amphibians, edited by P. M. Narins , A. S. Feng , R. R. Fay , and A. N. Popper , 221–249. Springer. 10.1007/978-0-387-47796-1_8.

[cne70100-bib-0105] Wild, J. M. 1995. “Convergence of Somatosensory and Auditory Projections in the Avian Torus Semicircularis, Including the Central Auditory Nucleus.” Journal of Comparative Neurology 358, no. 4: 465–486. 10.1002/cne.903580402. 7593743

[cne70100-bib-0106] Wild, J. M. , N. O. E. Krützfeldt , and M. Fabiana Kubke . 2010. “Connections of the Auditory Brainstem in a Songbird, *Taeniopygia guttata*. III. Projections of the Superior Olive and Lateral Lemniscal Nuclei.” Journal of Comparative Neurology 518, no. 11: 2149–2167. 10.1002/cne.22325. 20394063 PMC3865895

[cne70100-bib-0107] Willis, K. L. , and C. E. Carr . 2017. “A Circuit for Detection of Interaural Time Differences in the Nucleus Laminaris of Turtles.” Journal of Experimental Biology 220, no. 22: 4270–4281. 10.1242/jeb.164145. 28947499 PMC5702041

[cne70100-bib-0108] Winer, J. A. , and C. E. Schreiner . 2005. The Inferior Colliculus. Springer. 10.1007/b138578.

[cne70100-bib-0109] Woolley, S. M. N. , and J. H. Casseday . 2004. “Response Properties of Single Neurons in the Zebra Finch Auditory Midbrain: Response Patterns, Frequency Coding, Intensity Coding, and Spike Latencies.” Journal of Neurophysiology 91, no. 1: 136–151. 10.1152/jn.00633.2003. 14523072

[cne70100-bib-0110] Woolley, S. M. N. , and J. H. Casseday . 2005. “Processing of Modulated Sounds in the Zebra Finch Auditory Midbrain: Responses to Noise, Frequency Sweeps, and Sinusoidal Amplitude Modulations.” Journal of Neurophysiology 94, no. 2: 1143–1157. 10.1152/jn.01064.2004. 15817647

[cne70100-bib-0111] Yan, K. , Y. Tang , and C. E. Carr . 2010. “Calcium‐Binding Protein Immunoreactivity Characterizes the Auditory System of *Gekko gecko* .” Journal of Comparative Neurology 518, no. 17: 3409–3426. 10.1002/cne.22428. 20589907 PMC3170861

[cne70100-bib-0112] Yu, X. , Y. Peng , A. Aowphol , L. Ding , S. E. Brauth , and Y. Tang . 2011. “Geographic Variation in the Advertisement Calls of *Gekko gecko* in Relation to Variations in Morphological Features: Implications for Regional Population Differentiation.” Ethology Ecology and Evolution 23, no. 3: 211–228. 10.1080/03949370.2011.566581.

